# The Differentially Expressed Genes Responsible for the Development of T Helper 9 Cells From T Helper 2 Cells in Various Disease States: Immuno-Interactomics Study

**DOI:** 10.2196/42421

**Published:** 2023-02-23

**Authors:** Manoj Khokhar, Purvi Purohit, Ashita Gadwal, Sojit Tomo, Nitin Kumar Bajpai, Ravindra Shukla

**Affiliations:** 1 Department of Biochemistry All India Institute of Medical Sciences Jodhpur Jodhpur India; 2 Department of Nephrology All India Institute of Medical Sciences Jodhpur Jodhpur India; 3 Department of Endocrinology and Metabolism All India Institute of Medical Sciences Jodhpur Jodhpur India

**Keywords:** Th9 cells, Th2 cells, autoimmune diseases, DEGs, interleukins

## Abstract

**Background:**

T helper (Th) 9 cells are a novel subset of Th cells that develop independently from Th2 cells and are characterized by the secretion of interleukin (IL)-9. Studies have suggested the involvement of Th9 cells in variable diseases such as allergic and pulmonary diseases (eg, asthma, chronic obstructive airway disease, chronic rhinosinusitis, nasal polyps, and pulmonary hypoplasia), metabolic diseases (eg, acute leukemia, myelocytic leukemia, breast cancer, lung cancer, melanoma, pancreatic cancer), neuropsychiatric disorders (eg, Alzheimer disease), autoimmune diseases (eg, Graves disease, Crohn disease, colitis, psoriasis, systemic lupus erythematosus, systemic scleroderma, rheumatoid arthritis, multiple sclerosis, inflammatory bowel disease, atopic dermatitis, eczema), and infectious diseases (eg, tuberculosis, hepatitis). However, there is a dearth of information on its involvement in other metabolic, neuropsychiatric, and infectious diseases.

**Objective:**

This study aims to identify significant differentially altered genes in the conversion of Th2 to Th9 cells, and their regulating microRNAs (miRs) from publicly available Gene Expression Omnibus data sets of the mouse model using in silico analysis to unravel various pathogenic pathways involved in disease processes.

**Methods:**

Using differentially expressed genes (DEGs) identified from 2 publicly available data sets (GSE99166 and GSE123501) we performed functional enrichment and network analyses to identify pathways, protein-protein interactions, miR-messenger RNA associations, and disease-gene associations related to significant differentially altered genes implicated in the conversion of Th2 to Th9 cells.

**Results:**

We extracted 260 common downregulated, 236 common upregulated, and 634 common DEGs from the expression profiles of data sets GSE99166 and GSE123501. Codifferentially expressed ILs, cytokines, receptors, and transcription factors (TFs) were enriched in 7 crucial Kyoto Encyclopedia of Genes and Genomes pathways and Gene Ontology. We constructed the protein-protein interaction network and predicted the top regulatory miRs involved in the Th2 to Th9 differentiation pathways. We also identified various metabolic, allergic and pulmonary, neuropsychiatric, autoimmune, and infectious diseases as well as carcinomas where the differentiation of Th2 to Th9 may play a crucial role.

**Conclusions:**

This study identified hitherto unexplored possible associations between Th9 and disease states. Some important ILs, including *CCL1* (chemokine [C-C motif] ligand 1), *CCL20* (chemokine [C-C motif] ligand 20), *IL-13*, *IL-4*, *IL-12A*, and *IL-9*; receptors, including *IL-12RB1*, *IL-4RA* (interleukin 9 receptor alpha), *CD53* (cluster of differentiation 53), *CD6* (cluster of differentiation 6), *CD5* (cluster of differentiation 5), *CD83* (cluster of differentiation 83), *CD197* (cluster of differentiation 197), *IL-1RL1* (interleukin 1 receptor-like 1), *CD101* (cluster of differentiation 101), *CD96* (cluster of differentiation 96), *CD72* (cluster of differentiation 72), *CD7* (cluster of differentiation 7), *CD152* (cytotoxic T lymphocyte–associated protein 4), *CD38* (cluster of differentiation 38), *CX3CR1* (chemokine [C-X3-C motif] receptor 1), *CTLA2A* (cytotoxic T lymphocyte–associated protein 2 alpha), *CTLA28*, and *CD196* (cluster of differentiation 196); and TFs, including *FOXP3* (forkhead box P3), *IRF8* (interferon regulatory factor 8), *FOXP2* (forkhead box P2), *RORA* (RAR-related orphan receptor alpha), *AHR* (aryl-hydrocarbon receptor), *MAF* (avian musculoaponeurotic fibrosarcoma oncogene homolog), *SMAD6* (SMAD family member 6), *JUN* (Jun proto-oncogene), *JAK2* (Janus kinase 2), *EP300* (E1A binding protein p300), *ATF6* (activating transcription factor 6), *BTAF1* (B-TFIID TATA-box binding protein associated factor 1), *BAFT* (basic leucine zipper transcription factor), *NOTCH1* (neurogenic locus notch homolog protein 1), *GATA3* (GATA binding protein 3), *SATB1* (special AT-rich sequence binding protein 1), *BMP7* (bone morphogenetic protein 7), and *PPARG* (peroxisome proliferator–activated receptor gamma, were able to identify significant differentially altered genes in the conversion of Th2 to Th9 cells. We identified some common miRs that could target the DEGs. The scarcity of studies on the role of Th9 in metabolic diseases highlights the lacunae in this field. Our study provides the rationale for exploring the role of Th9 in various metabolic disorders such as diabetes mellitus, diabetic nephropathy, hypertensive disease, ischemic stroke, steatohepatitis, liver fibrosis, obesity, adenocarcinoma, glioblastoma and glioma, malignant neoplasm of stomach, melanoma, neuroblastoma, osteosarcoma, pancreatic carcinoma, prostate carcinoma, and stomach carcinoma.

## Introduction

CD4^+^ T helper (Th) cells have been classified into different subsets based on the cytokine profile that each subset secretes and their distinct role in regulating immunity and inflammation. Previous studies have shown that immune cells play a role in various metabolic [1-3] and infectious [3-7] diseases. Th9 cells are a subset of CD4^+^ Th cells that develop from naïve T cells and release interleukin (IL)-9. The generation of Th9 cells from naïve Th0 cells requires a Th2 state as an intermediate. While both Th2 and Th9 cells express *PU.1* (spleen focus forming virus [SFFV] proviral integration oncogenes), *IRF4* (interferon regulatory factor 4), and *GATA3* (GATA binding protein 3), the latter have upregulated expression of *IRF4* and suppressed *PU.1.* The Th2 cells, generated during Th0 cell differentiation, further evolve into Th9 cells in the presence of activated *Smad3/Smad4* and *IRF4* pathways. The prolonged transforming growth factor beta (*TGFβ*) stimulation transforms the Th2 cells into Th9 cells and alters the cytokine secretion pattern from an *IL-4*–dominant phenotype to an *IL-9*–dominant one [8]. Th9 cells produce *IL-9*, which is crucial in regulating autoimmune and allergic reactions [9]. Various other cytokines also affect the development of Th9 cells and *IL-9* production. *IL-23* inhibits *IL-9* production, whereas *IL-1* and *IL-33* stimulate the production of *IL-9* in T cells [10,11]. Similarly, *IL-25* stimulates the release of *IL-9* from T cells [12]. In addition, costimulatory receptors, such as *OX40,* have been found to be a stimulant for the development of Th9 cells [13]. Thus, the development of Th9 cells is a result of integrating multiple positive and negative signals in the form of cytokines and costimulation from surface receptors.

Th9 cells can manifest differently in various diseases. Th9 cells have been demonstrated to incite allergic airway disease [14]. Th9 cells have also been implicated in tumor immunity [10]. Interestingly, the evolution of Th2 to Th9 cells does influence the pathophysiology of multiple diseases. The nitric oxide–mediated airway inflammation has been attributed to the inducing effect of nitric oxide on the development of Th9 cells [15]. The tricarboxylic acid cycle metabolite succinate stimulates Th9 cell differentiation and leads to Th9 cell–mediated tumor regression. Similarly, Th9 differentiation resulting from *IL-35* stimulation accentuates the inflammatory process and leads to an immunoglobulin (Ig) class switch toward IgG4 in IgG4-related diseases [16].

Unfortunately, the experimental approach to Th9 cells has been riddled with difficulty, because a selective deficiency model for Th9 lineage has not yet been defined. In addition, factors needed to develop Th9 cells such as *IL-4* and *IRF4* are required to develop other Th subsets [17]. Our study aimed to compare the transcriptome of Th2 and Th9 cells to identify the pattern of changes in the expression of various genes when the Th2 cells get differentiated into Th9 cells. We also aimed to assess these genes, which are markedly altered in the transition of Th2 to Th9 cells, in various other diseases to enlist the possible diseases in which Th9 cells may play a crucial role.

## Methods

### Expression Profiling: Gene Expression Omnibus Assay to Data Mining for Th2 to Th9 Cells Differentiation

**Figure 1:**
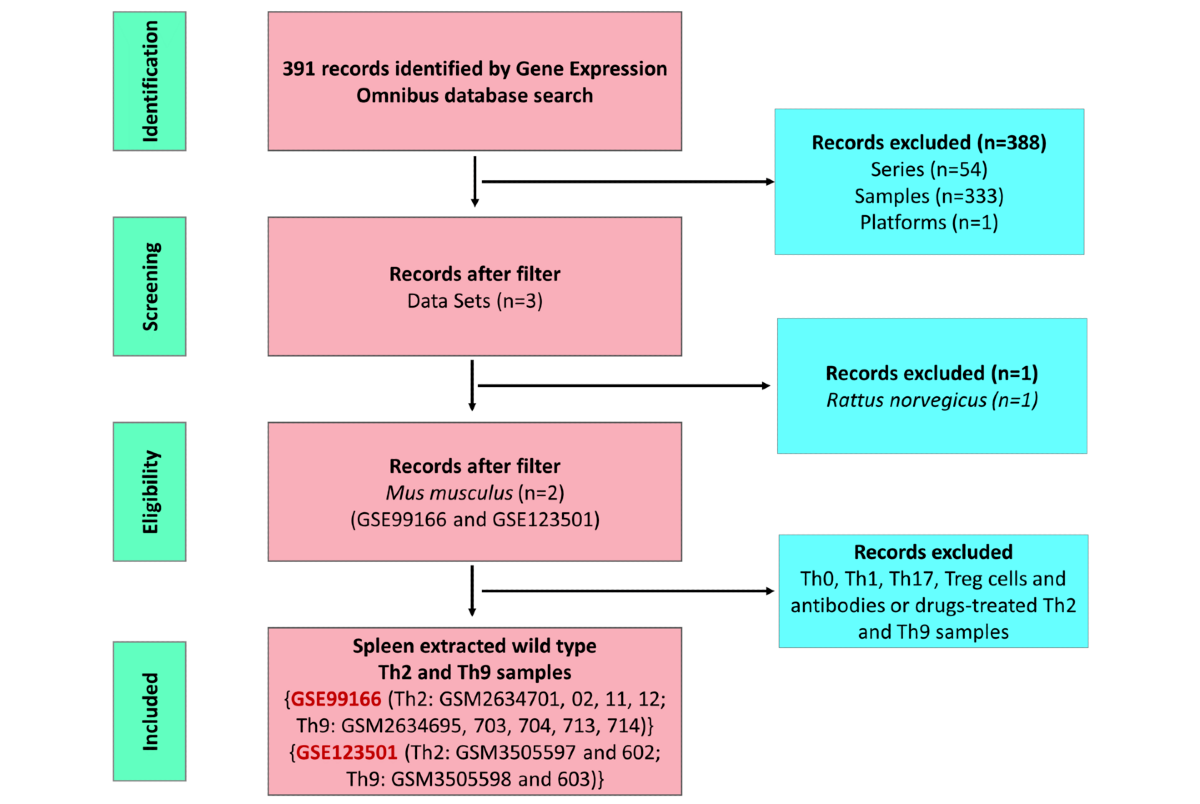
Flow diagram illustrating the data collection process and the number of data sets considered for inclusion. Th: T helper; Treg: T regulatory cell.

We performed a search in the Gene Expression Omnibus (GEO) database using several keywords, including “Healthy Control,” “Wild Type,” “Mice,” “*Mus musculus*,” “Th9,” “Th2,” and “Expression profiling by array” from January 1, 2012, to December 17, 2020, and selected 2 gene series expressions (GSEs) data for further study: GSE99166 and GSE123501. GSE99166 contained 4 samples of Th2 wild-type cells (GSM2634701, GSM2634702, GSM2634711, and GSM2634712) and 5 samples of Th9 wild-type cells (GSM2634695, GSM2634703, GSM2634704, GSM2634713, and GSM2634714) from the spleen. GSE123501 contained 2 samples of Th2 wild-type cells (GSM3505597 and GSM3505602) and another 2 samples of Th9 wild-type cells (GSM350598 and GSM3505603) from the spleen (Figure 1).

### Assortment and Identification of Codifferentially Expressed Messenger RNAs From the Spleen (2 Different) Data Sets

**Figure 2:**
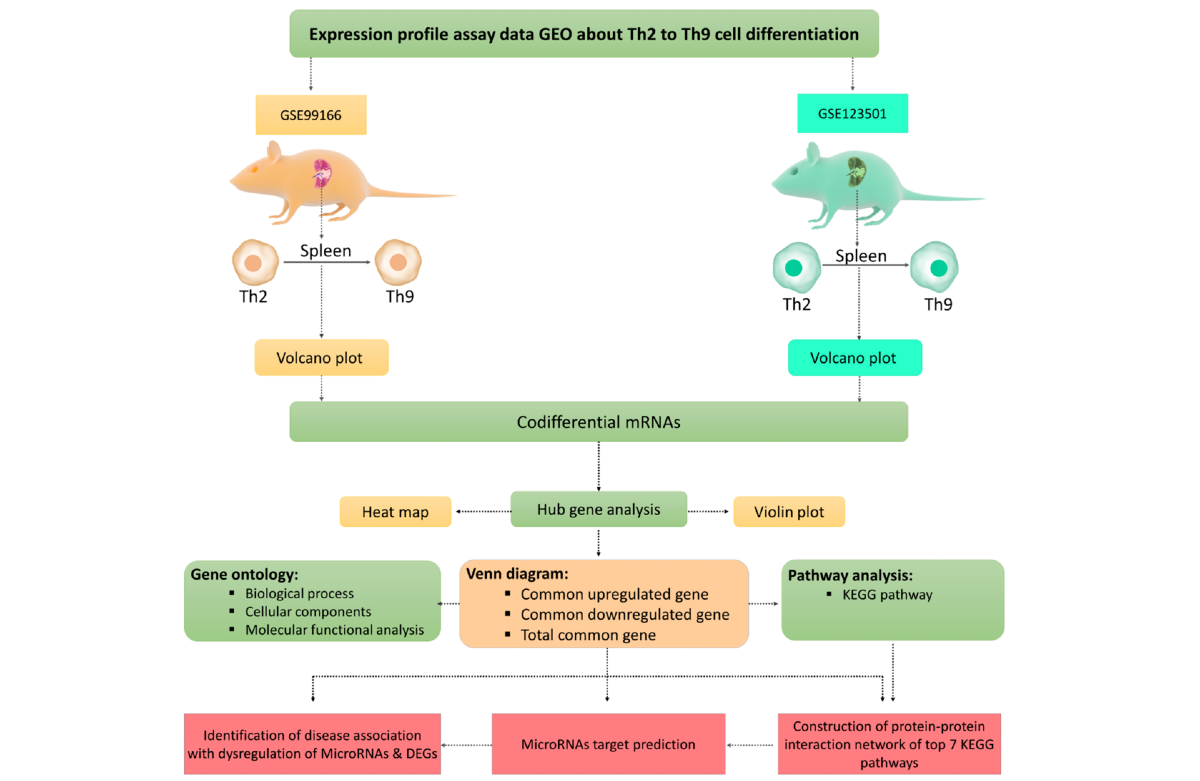
Flowchart of the data processing and analysis for Th2 to Th9 cells differentiation. DEG: differentially expressed gene; KEGG: Kyoto Encyclopedia of Genes and Genomes; mRNA: messenger RNA; Th: T helper.

The differentially expressed genes (DEGs) were obtained from the 13 samples of 2 different data sets (GSE99166 and GSE123501) using the GREIN (GEO RNA-seq Experiments Interactive Navigator) platform (BD2K-LINCS Data Coordination and Integration Center). This interactive online web tool analyses GEO RNA-seq data [18]. The DEGs extracted from the data sets comprised genes from Th2 and Th9 cells. As we wanted to assess the alteration of genes during the conversion of Th2 to Th9 cells, the analysis was performed with DEGs of Th2 cells as the standard to which DEGs of Th9 cells were compared. The workflow for the data processing and analysis is portrayed in Figure 2.

The DEGs were considered upregulated when the expression of genes in Th9 cells was higher than that in Th2 cells. The cutoff for the selection was kept at *P*<.05, and overlapping DEGs between 2 data sets (GSE99166 and GSE123501) on comparison of Th2 and Th9 cells were identified by the Venn diagram tool [19,20]. In addition, the common upregulated, downregulated, and oppositely regulated DEGs of these 2 data sets (GSE99166 and GSE123501) were identified. The fold change expression distribution was visualized by a heat map and violin plot using the Linear Models for the Microarray Data (limma) Package of R (R Foundation for Statistical Computing) and Orange Data Mining (University of Ljubljana) [21,22].

### Functional Enrichment of Gene Ontology for Common, Regulated DEGs

The codifferential genes were divided into 3 parts, namely, (1) common upregulated, (2) common downregulated, and (3) common, oppositely regulated. The top ranked ontological features of all DEGs were analyzed with STRING. The Gene Ontology (GO) terms included the following 3 categories: biological processes, cellular components, and molecular functions. The significant GO terms regulating genes are presented in a radar graph with a negative log10 (false discovery rate). We defined *P*<.05 as a significant value.

### Kyoto Encyclopedia of Genes and Genomes Pathway Analysis of Top Ranked Significant, Common, Regulated DEGs

We searched the functionally significant Kyoto Encyclopedia of Genes and Genomes (KEGG) pathways for top ranked significantly altered DEGs using the STRING and WikiPathways databases. We identified important genes participating in each pathway, and selected the top 7 pathways based on negative log10 (false discovery rate) and P values (<.05) that were important for further study.

### Genes Assortment and Construction of a Protein-Protein Interaction Network of the Top Enriched Pathways

We downloaded the complete gene list of the top ranked 7 individual pathways with an interaction network from the KEGG database. We revisualized and constructed the pathway with the help of Cytoscape (Cytoscape Team/Institute for Systems Biology; an open-source software platform for visualizing complex networks and integrating these with any type of attribute data) [23] and marked the DEGs that play a significant role in the differentiation of Th2 to Th9 cells.

### Identification of Top Regulatory MicroRNAs Involved in the Th2 to Th9 Differentiation Pathways

The top 10 microRNAs (miRs) that targeted the hub genes were predicted by the well-established miR target prediction database miRNet version 22.0 [24], with special emphasis on the selected organism. Default values for the degree of interaction and betweenness were selected. Common miRs and their targeted messenger RNAs (mRNAs) of all groups were sorted by the Venn diagram.

### Construction of a Gene-Disease–Based Genomic Pathway Interaction Network

The DEGs that were identified to play a significant role in Th2 to Th9 differentiation were further analyzed for their involvement in various pathways pertaining to specific diseases using DisGeNET (IBI Group) [25], a discovery platform that describes genes, transcription factors (TFs), chemokines, and IL in association with various specific diseases.

### Ethical Considerations

The study was approved by the Institutional Ethics Committee of All India Institute of Medical Sciences (AIIMS) Jodhpur (certificate reference number AIIMS/IEC/2019-20/792).

## Results

### Assortment of Significant DEGs in the Differentiation of Th2 to Th9 Cells

**Figure 3:**
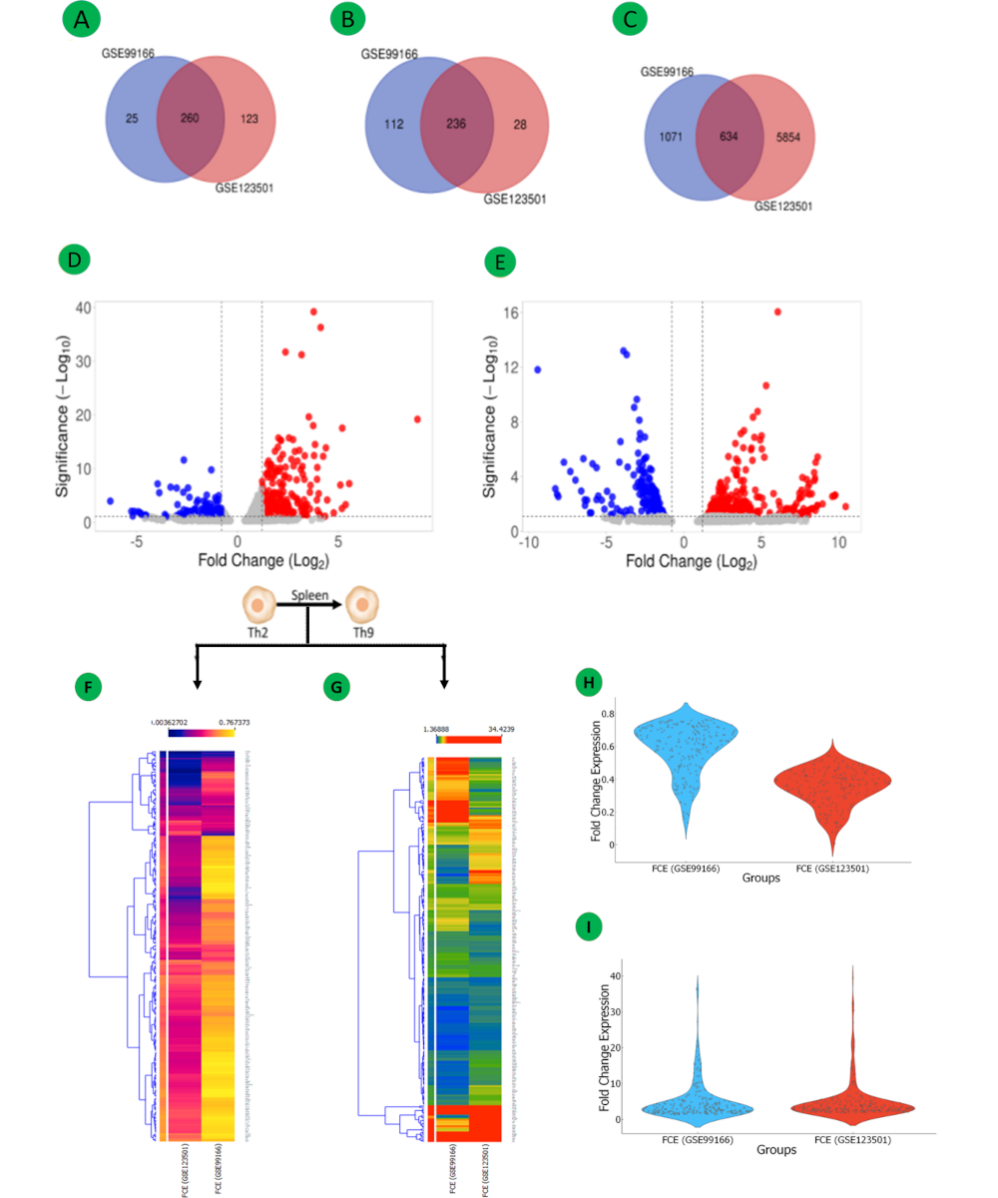
Differential mRNA expression of the 2 data sets (GSE99166 and GSE123501) for Th2 to Th9 differentiation: Venn diagrams (A-C) of the 3 data sets' total common, downregulated, and upregulated DEGs; volcano plot for the data sets GSE99166 (D) and GSE123501 (E); and heat maps of common downregulated (F) and common upregulated (G) DEGs extracted from the data sets consisting of genes from Th2 and Th9 cells. FCE levels are displayed in ascending order from blue to yellow. Violin plots (H and I) showing FCE distribution of both data sets for common upregulated and common downregulated DEGs. DEG: differentially expressed gene; FCE: fold change expression; mRNA: messenger RNA; Th: T helper.

The *Mus musculus* (C57BL/6) mRNA expression profiles of GSE99167 and GSE123501, which were selected for this study, included the expression profiles of Th2 and Th9 cells obtained from the spleen. We extracted and compared mice spleen samples from 2 different studies to identify genes that are involved in the differentiation of Th2 to Th9 cells. In both groups, 254 common mRNAs were identified, and 634 common DEGs were identified, of which 236 were downregulated and 260 were upregulated. We performed a quality assessment of the selected samples for our expression profiles (Figures 3A-3I; see Tables S1 and S2 in Multimedia Appendix 1, and Multimedia Appendix 2 for larger version of figures).

### Identification and Assortment of Codifferentially Expressed ILs, Cytokines, Receptors, and TFs

**Figure 4:**
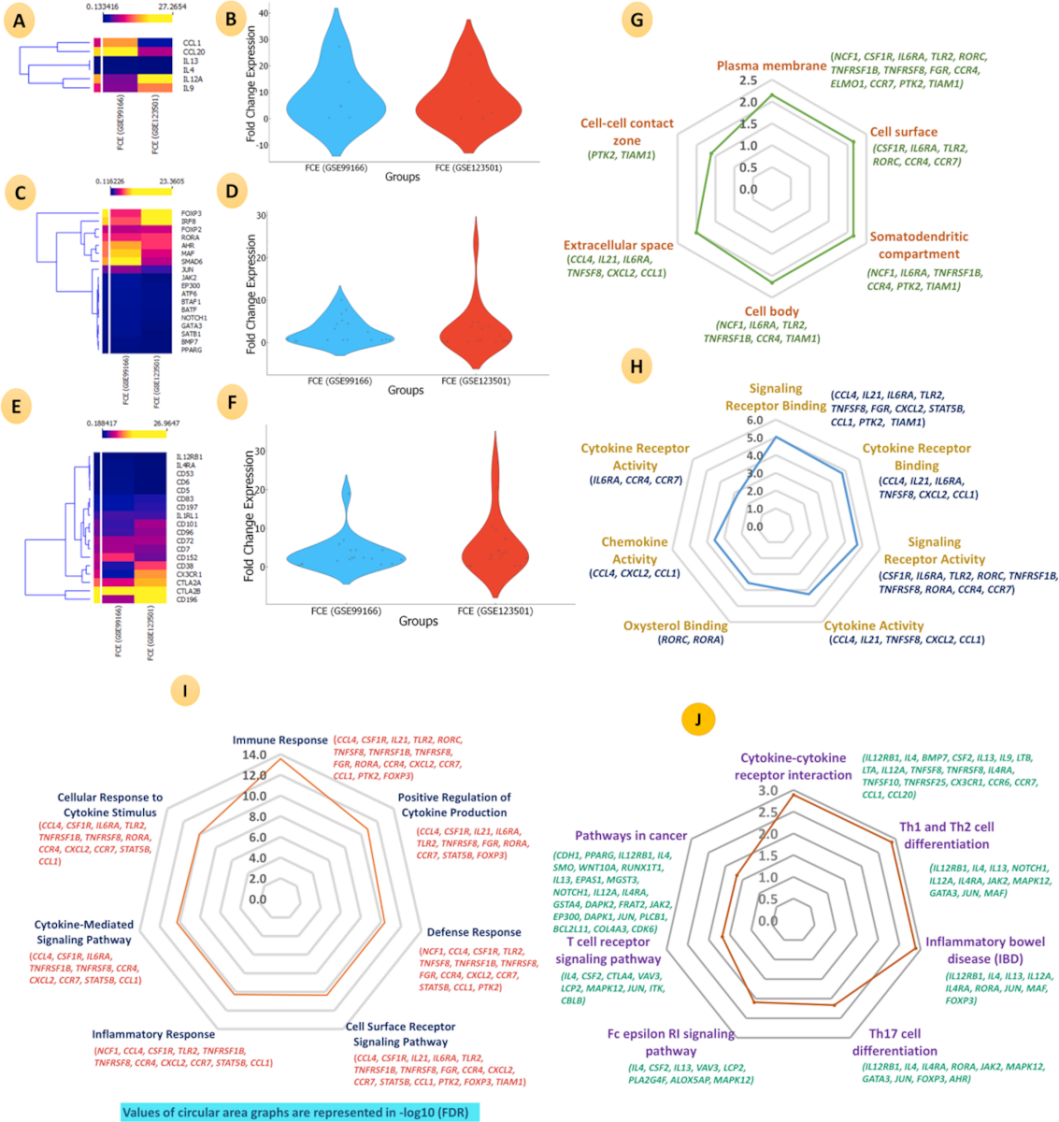
Differential mRNA expression of the 2 data sets (GSE99166 and GSE123501) for Th2 to Th9 differentiation: (A, B) interleukin, cytokines/chemokines; (C, D) immune regulating transcription factor; (E, F) immune receptor heat maps and violin plot of all, downregulated, and upregulated DEGs; (G, J) enrichment analysis of common DEGs; (G) Gene Ontology cellular component terms; (H) Gene Ontology molecular function terms; (I) Gene Ontology biological process terms; and (J) the KEGG pathway. The radar plot or circular area graph values are represented in the -log10 (FDR). DEG: differentially expressed gene; FCE: fold change expression; FDR: false discovery rate; KEGG: Kyoto Encyclopedia of Genes and Genomes; mRNA: messenger RNA; Th: T helper.

Our analysis identified genes encoding various ILs and receptors whose differential expression may determine the differentiation of Th2 to Th9 cells. Some important ILs identified were *CCL1* (chemokine [C-C motif] ligand 1), *CCL20* (chemokine [C-C motif] ligand 20), *IL-13, IL-4, IL-12A,* and *IL-9.* The important receptors identified in our analysis were *IL-12RB1, IL-4RA* (interleukin 4 receptor alpha), *CD53* (cluster of differentiation 53), *CD6* (cluster of differentiation 6), *CD5* (cluster of differentiation 5), *CD83* (cluster of differentiation 83), *CD197* (cluster of differentiation 197), *IL-1RL1* (interleukin 1 receptor-like 1), *CD101* (cluster of differentiation 101), *CD96* (cluster of differentiation 96), *CD72* (cluster of differentiation 72), *CD7* (cluster of differentiation 7), *CD152* (cytotoxic T lymphocyte–associated protein 4), *CD38* (cluster of differentiation 38), *CX3CR1* (chemokine [C-X3-C motif] receptor 1), *CTLA2A* (cytotoxic T lymphocyte–associated protein 2 alpha), *CTLA28,* and *CD196* (cluster of differentiation 196)*.* In addition, the differential expression of various TFs such as *FOXP3* (forkhead box P3), *IRF8* (interferon regulatory factor 8), *FOXP2* (forkhead box P2), *RORA* (RAR-related orphan receptor alpha), *AHR* (aryl-hydrocarbon receptor), *MAF* (avian musculoaponeurotic fibrosarcoma oncogene homolog), *SMAD6* (SMAD family member 6), *JUN* (Jun proto-oncogene), *JAK2* (Janus kinase 2), *EP300* (E1A binding protein p300), *ATF6* (activating transcription factor 6), *BTAF1* (B-TFIID TATA-box binding protein associated factor 1), *BAFT* (basic leucine zipper transcription factor), *NOTCH1* (neurogenic locus notch homolog protein 1), *GATA3, SATB1* (special AT-rich sequence binding protein 1), *BMP7* (bone morphogenetic protein 7), and *PPARG* (peroxisome proliferator–activated receptor gamma) may influence the differentiation of Th2 to Th9 cells. The expression of the aforementioned immune regulators is represented by a heat map and Venn diagram in Figures 4A-4F (also see Tables S3-S5 in Multimedia Appendix 1, and Multimedia Appendix 2 for larger version of figures).

### Functional Enrichment and KEGG Pathway Analysis of DEGs Involved in the Transition of Th2 to Th9 Cells

A GO analysis of DEGs classified them into 3 functional classes (Figures 4G-4I; see Multimedia Appendix 2 for larger versions of figures): cellular component, biological process, and molecular function.

The enrichments for the 3 DEG classes with significantly altered expression are shown in Tables S6-S8 in Multimedia Appendix 1. In the KEGG pathway enrichment analysis, the identified genes were enriched in various KEGG pathways such as cytokines-cytokines interaction, Th1 and Th2 cell differentiation, inflammatory bowel disease (IBD), Th17 cell differentiation, the Fc epsilon RI signaling pathway, the T-cell receptor signaling pathway, and pathways in cancer (Figure 4J and Tables S9 and S10 in Multimedia Appendix 1; see Multimedia Appendix 2 for larger version of figures).

### Construction of the Protein-Protein Interaction Network of DEGs Involved in the Transition of Th2 to Th9 Cells

**Figure 5:**
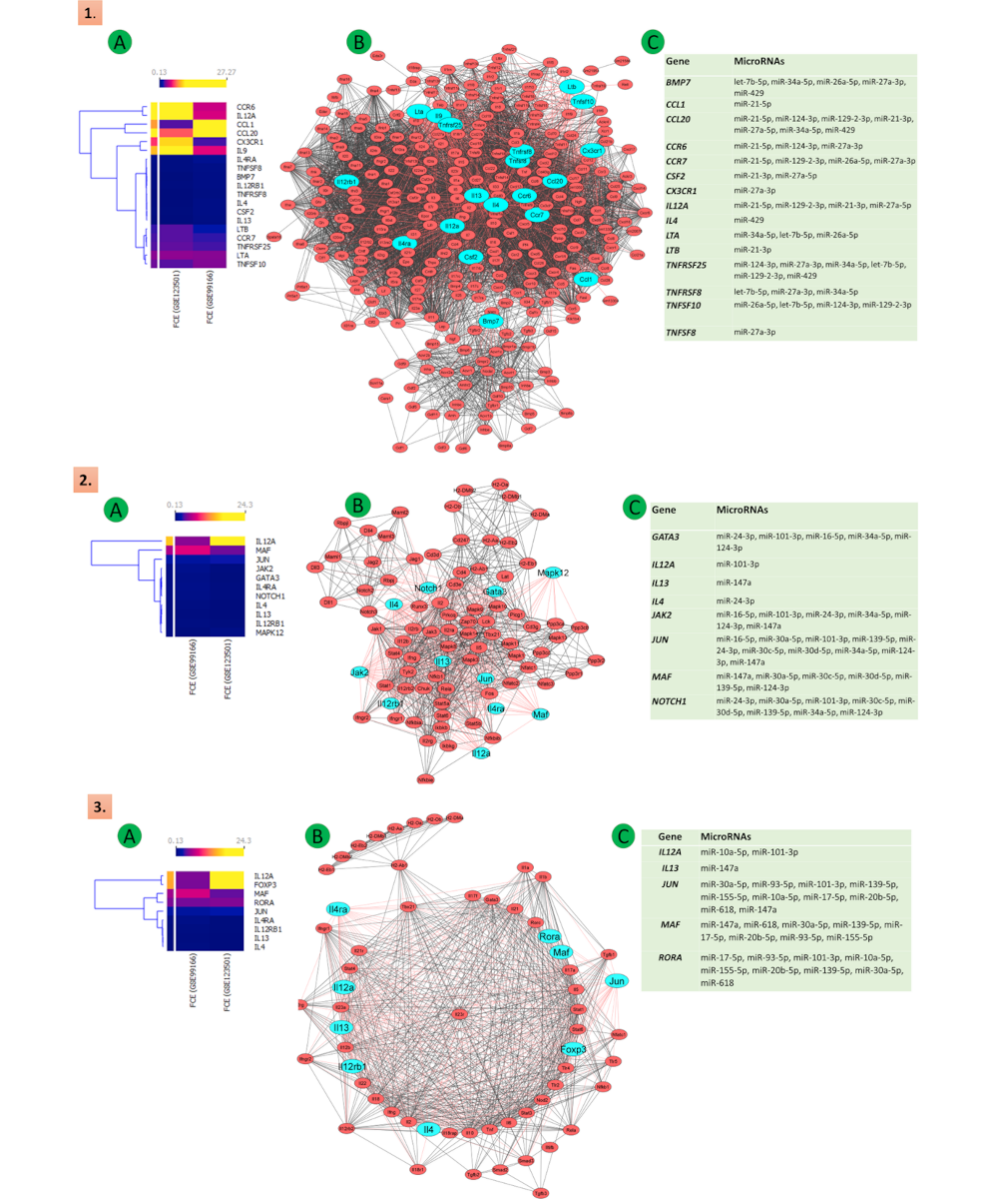
Illustration of the Th2 to Th9 differentiation, mainly the 7 pathways involved in this regulatory mechanism: (A) heat maps expression (the upper section of the heat map shows FCE values represented by varying color densities); (B) PPIs networks (top significant DEGs of the network are illustrated in cyan); (C) common posttranscriptional regulatory microRNA pathways—(1) cytokine-cytokine receptor interaction, (2) Th1 and Th2 cell differentiation, and (3) inflammatory bowel disease. FCE: fold change expression; PPI: protein-protein interaction; Th: T helper.

**Figure 6:**
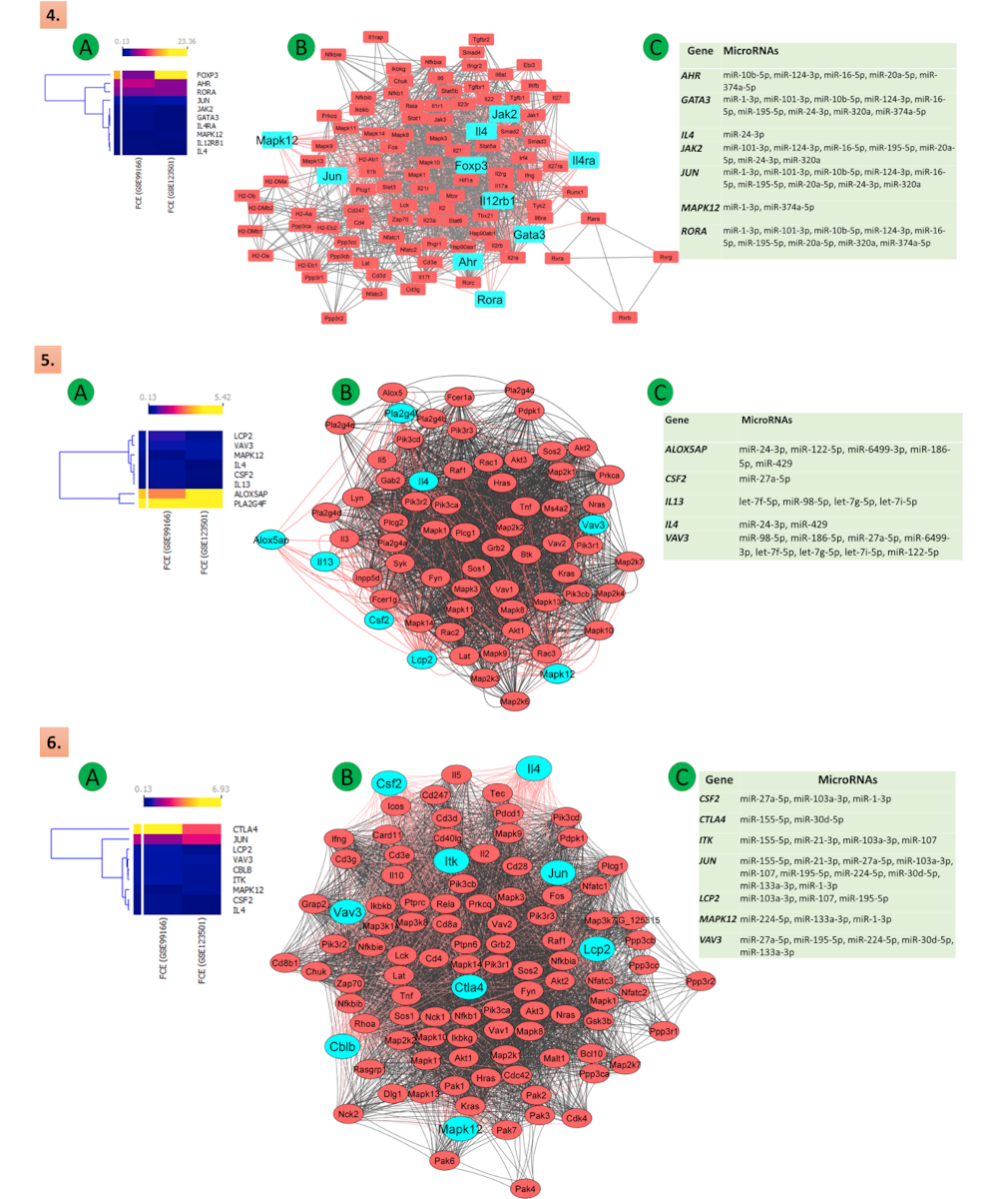
Illustration of the Th2 to Th9 differentiation, mainly the 7 pathways involved in this regulatory mechanism: (A) heat maps expression (the upper section of the heat map shows FCE values represented by varying color densities); (B) PPIs networks (top significant DEGs of the network are illustrated in cyan); (C) common posttranscriptional regulatory microRNA pathways—(4) Th17 cell differentiation, (5) Fc epsilon and RI pathway, (6) T-cell receptor signaling pathway. DEG: differentially expressed gene; FCE: fold change expression; PPI: protein-protein interaction; Th: T helper.

**Figure 7:**
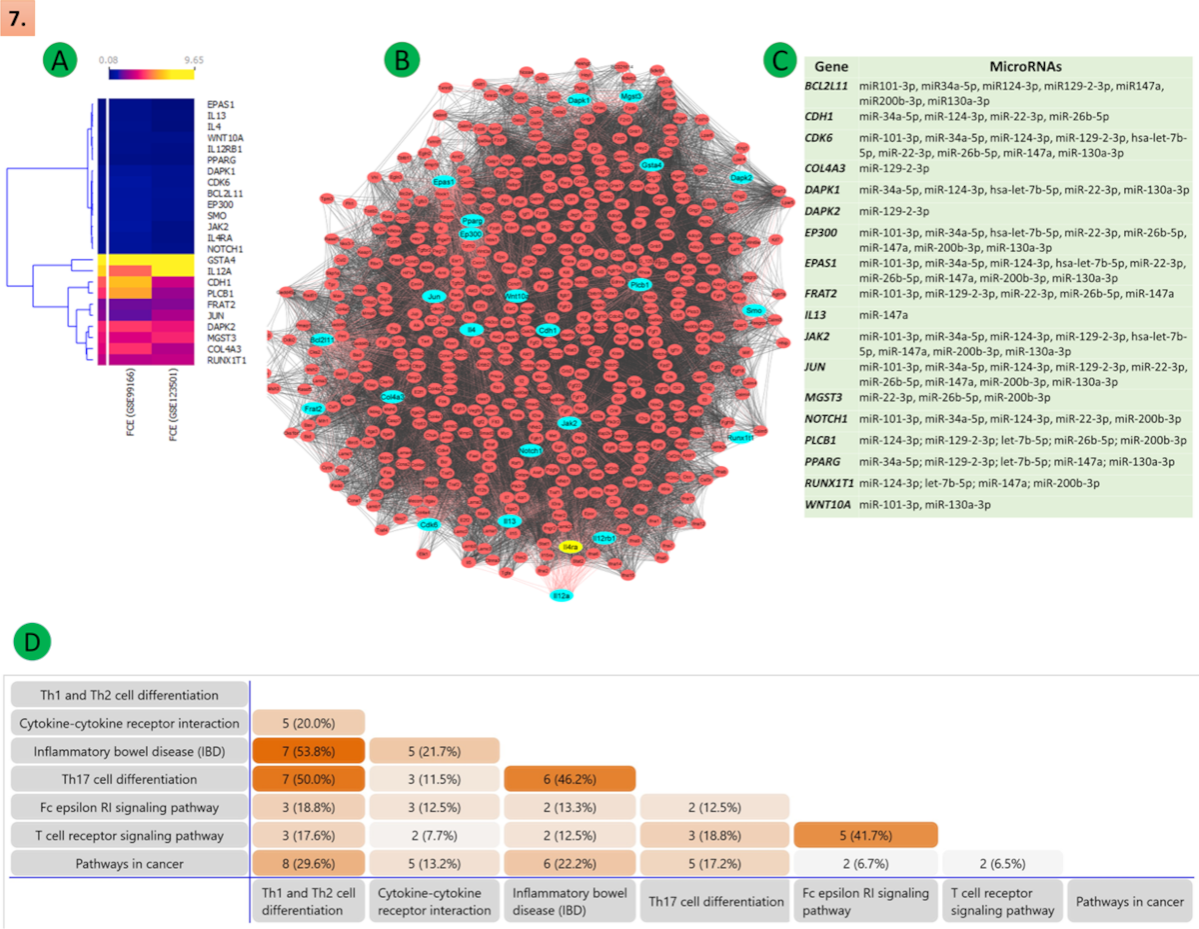
Illustration of the Th2 to Th9 differentiation, mainly the 7 pathways involved in this regulatory mechanism: (A) heat maps expression (the upper part of the heat map shows FCE values represented by varying color densities); (B) PPIs networks (top significant DEGs of the network are presented in cyan); (C) common posttranscriptional regulatory microRNA pathways; (D) gene or DEGs similarity in regulatory pathways. DEG: differentially expressed gene; FCE: fold change expression; PPI: protein-protein interaction; Th: T helper.

We downloaded the complete protein-protein interaction (PPI) network of the identified KEGG pathways from the KEGG database. The Cytoscape software was used for the construction of the network. The significantly altered DEGs of cytokines, chemokines, receptors, and TFs were highlighted in the respective networks. Our analysis of the KEGG pathway enrichment and PPI network demonstrated that the genes that had a significantly altered expression in Th9 cells when compared with Th2 cells also played a significant role in other immune regulating pathways. These affected pathways were mainly involved in cytokines-cytokines interaction, Th1 and Th2 differentiation, CTLA4 (cytotoxic T lymphocyte–associated protein 4) regulation, T-cell receptor signaling, Fc epsilon signaling, Th17 cell differentiation, IBD, and cancer. The concurrent presence of these genes in the aforementioned pathways highlights the significance of the differentiation of Th2 to Th9 in diseases where these pathways are affected. The role of the identified DEGs in these pathways and their interaction with other genes has been depicted in Figures 5-7. See Multimedia Appendix 2 for larger images.

### Assessment of Gene Similarity in Pathways Identified in the KEGG Pathway Enrichment Analysis

We performed a gene similarity analysis to find similar genes in all the 7 KEGG pathways identified with the help of the Venn diagram and calculate the percentage of similarity among the genes that were altered. We observed that 7/13 (54%) genes were similar between the “Th1 and Th2 cell differentiation” and “IBD” pathways, whereas 7/14 (50%) genes were similar between the “Th1 and Th2 cell differentiation” and “Th17 cell differentiation” pathways (Figure 7A; see Multimedia Appendix 2 for larger images).

### Prediction of miRs That Target the DEGs Involved in the Transition of Th2 to Th9 cells

To explore the posttranscriptional regulation of the identified DEGs, we predicted the miRs that could target the identified DEGs. We identified the following 53 common miRs that could target the DEGs listed in our analysis: let-7b-5p, let-7f-5p, let-7g-5p, let-7i-5p, miR-1-3p, miR-101-3p, miR-103a-3p, miR-107, miR-10a-5p, miR-10b-5p, miR-122-5p, miR-124-3p, miR-129-3p, miR-130a-3p, miR-133a-3p, miR-139-5p, miR-147a, miR-155-5p, miR-16-5p, miR-17-5p, miR-186-5p, miR-195-5p, miR-200b-3p, miR-20a-3p, miR-20a-5p, miR-20b-5p, miR-21-3p, miR-21-5p, miR-22-3p, miR-224-5p, miR-24-3p, miR-26a-5p, miR-26b-5p, miR-27-5p, miR-27a-3p, miR-302a, miR-30a-5p, miR-30c-5p, miR-30d-5p, miR-320a, miR-34-5p, miR-374-5p, miR-426, miR-429, miR-618, miR-6499-3p, miR-93-5p, miR-98-5p, miR-103a-3p, miR-139-5p, miR-147a, miR-195-5p, and miR-27a-5p.

### Identification of Diseases Associated With Dysregulation of the Identified miRs and DEGs

**Figure 8:**
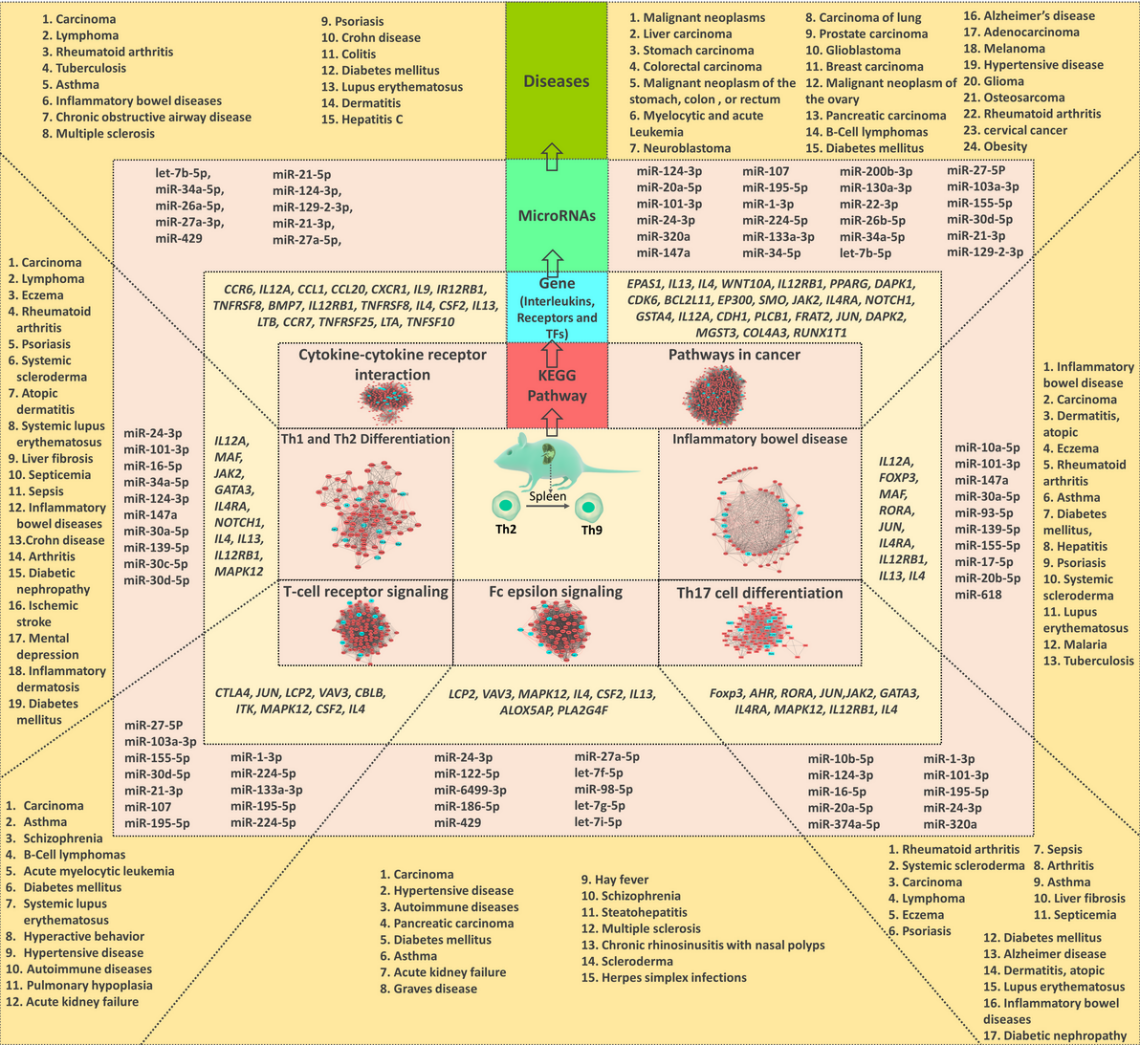
The diagram illustrates the molecular regulatory process of Th2 to Th9 differentiation in mouse spleen. This regulatory mechanism is regulated by 7 major pathways controlled by some specific immune regulatory transcription factors, receptors, and cytokines/chemokines. The standard and specific microRNAs play a crucial role in the posttranscriptional regulatory mechanism. These 7 pathways regulate DEGs misexpression involved in some critical diseases. DEG: differentially expressed gene; KEGG: Kyoto Encyclopedia of Genes and Genomes; Th: T helper.

Subsequent to the identification of pathways affected as a result of the alteration of DEGs found in our analysis, we further searched for possible diseases whose pathogenesis is affected by alterations in these pathways. We listed the diseases where alterations in the aforementioned 7 pathways have already been documented, and these were as follows: metabolic diseases (eg, diabetes mellitus, diabetic nephropathy, hyperactive behavior, hypertensive disease, ischemic stroke, steatohepatitis, liver fibrosis, obesity), allergic and pulmonary diseases (eg, asthma, chronic obstructive airway disease, chronic rhinosinusitis, nasal polyps, pulmonary hypoplasia, hay fever), carcinomas (eg, acute leukemia and myelocytic leukemia, B-cell lymphomas, lymphoma, adenocarcinoma, breast carcinoma, carcinoma of the lung, cervical cancer, colorectal carcinoma, glioblastoma and glioma, liver carcinoma, malignant neoplasm of the stomach, melanoma, neuroblastoma, osteosarcoma, pancreatic carcinoma, prostate carcinoma, stomach carcinoma), neuropsychiatric disorders (eg, mental depression, schizophrenia, Alzheimer disease), autoimmune diseases (eg, Graves disease, Crohn disease, colitis, psoriasis, systemic lupus erythematosus [SLE], systemic scleroderma, rheumatoid arthritis, multiple sclerosis [MS], IBD, atopic dermatitis, eczema), and infectious diseases (eg, sepsis, septicemia, tuberculosis, hepatitis, herpes simplex infections, malaria; Figure 8).

## Discussion

### Principal Findings

In this study, we compared 2 different data sets (GSE99166 and GSE123501) that have compared the mRNA expression in Th2 and Th9 cells. We identified common DEGs that have significantly altered expression between Th2 and Th9 cells from these 2 data sets. Sequential assessment of the DEGs and miRs that had significantly altered expression between Th2 and Th9 cells allows to identify disease states that affect the differentiation process. Although this analysis does not answer whether differentiation of Th2 to Th9 is the cause or the effect of the disease state, it does unravel the possibility of hitherto unknown associations between various diseases and the process of differentiation of Th2 to Th9 cells. Our analysis indicates that differentiation of Th2 to Th9 may play a crucial role via the alteration of DEGs (Table 1) and miRs (Table 2) in various metabolic diseases, allergic and pulmonary diseases, carcinomas, neuropsychiatric disorders, autoimmune diseases, and infectious diseases. In concordance with the existing literature, it was revealed that Th9 cells might play a major role in erythematosus, MS, IBDs, and psoriasis. The role of Th9 cells in autoimmune disease has already been explored in multiple studies [26], including in Graves disease [27], Crohn disease [28-30], psoriasis [31], SLE [32-35], systemic scleroderma [36], rheumatoid arthritis [37-40], MS [26,36,41,42], IBD [26,29,30,43], and atopic dermatitis/eczema [44], which have demonstrated an increased level of differentiation of Th2 to Th9 cells. Th9 cells and *IL-9* have been observed in peripheral blood mononuclear cells and synovial fluid from patients with rheumatoid arthritis. Toll-like receptor 2 (*TLR2*) stimulates naïve CD4^+^ T cells for *IL-9* secretion and Th9 differentiation by increasing the expression of TFs *BATF* and *PU.1*. *TLR2* activation results in increased expression of *IL-33* and its receptor ST2, augmenting *IL-9* gene expression and Th9 cell development [45]. Similarly, in patients with SLE, Th9 cell differentiation is suppressed by repression of *IRF4* expression [46]. Although the role of Th9 has been explored in experimental models of MS and IBD, there is insufficient evidence regarding its role in humans. Th9 cells have been shown to play a pathogenic role in experimental autoimmune encephalomyelitis, an animal model of MS [47]. However, only limited studies have assessed Th9 cells in human patients with MS. The skin toxicity of Th9 cells makes them a crucial link in the pathophysiology of multiple skin diseases [48]. Our study highlights the possibility of Th9 playing a crucial role in the pathophysiology of various autoimmune skin diseases such as eczema, atopic dermatitis, psoriasis, and dermatitis. A predominant expression of *IL-9* from Th9 cells was observed to be a characteristic immunologic signature in psoriatic arthritis [49]. Similarly, *IL-9* and *PU.1* gene expressions in atopic dermatitis were higher and associated with disease severity [50]. In addition, the Th9 cell percentage in patients with atopic dermatitis correlated with serum IgE levels, highlighting the link between allergy and the development of Th9 cells [51]. Our in silico analysis further reiterated the involvement of Th9 in various autoimmune pathways. The involvement of *IL-9* and Th9 cells in allergic response can also be seen in other diseases. One such allergic disease in which Th9 cells have been recently explored is asthma. Patients with allergic asthma have increased peripheral blood Th9 cells and elevated levels of serum *IL-9* [51]. *SGK1* (serum/glucocorticoid regulated kinase 1) has been shown to enhance the differentiation of Th9 by modulating the nuclear factor kappa B (*NF-κB*) signaling pathway in patients with asthma [52]. The activation of *MAPK* (mitogen-activated protein kinase) has also been attributed to the activation of Th9 cells in mice models of asthma [53]. Interestingly, *IL-9* and *IL-13* have been elevated in patients with chronic obstructive airway disease compared with asthma [54]. However, so far, the Th9 cells have not been explored for their significance in the pathophysiology of chronic obstructive pulmonary disease. Interestingly, apart from asthma, our in silico analysis highlighted chronic obstructive airway disease, tuberculosis, and chronic rhinosinusitis with nasal polyps as major airway diseases in which Th9 cells may play a crucial role. Our findings are in sync with the study of Ye et al [55], which demonstrated tuberculous pleural effusion to be chemotactic for Th9 cells, while pleural mesothelial cells in tuberculosis stimulated the Th9 cell differentiation. This in silico analysis also highlights the possible role of Th9 in neuropsychiatric diseases. Very few studies have explored the role of Th9 in neuropsychiatric disorders. Saresella et al [56] have demonstrated an increase in the activity of Th9 lymphocytes, while postthymic maturation pathways showed an accumulation of differentiated effector T lymphocytes (CD4^+^). In Alzheimer disease, schizophrenia, and multiple-episode schizophrenia, although *IL-9* has been elevated, limited studies have been performed to assess the role of Th9 cells in the pathophysiology of the diseases [56,57]. In addition to the aforementioned diseases, this study identified malignancies as one of the disease states that could be affected by the development of Th9 cells. The role of Th9 cells in modulating immunity in cancer has been widely explored. Th9 cells contribute to antitumor immunity by enhancing the recruitment and activation of mast cells, natural killer cells, CD8 T cells, and dendritic cells in the tumor microenvironment. The antitumor effect of Th9 cells has been documented in various animal studies. Lu et al [58] have demonstrated the protective effects of *IL-9* and Th9 on tumor development. The tumor-specific Th9 cells promoted the activation of CD8^+^ cytotoxic T lymphocytes by recruiting dendritic cells into tumor tissues and subsequently presenting tumor antigens in tumor-draining LNs. Th9 cells in tumor tissues mount an inflammatory response via CTL in a *CCL20/CCR6* (chemokine [C-C motif] receptor 6)-dependent manner [59,60]. Wang et al [61] also demonstrated that Th9-enriched CD4^+^ T cells significantly increased the expansion of activated CD8^+^ T cells in a manner that was dependent on the expression of *IL-9R* (interleukin 9 receptor)*.* Th9 thus seems to enhance antitumor immune response through T-cell cytotoxicity and play a crucial role in controlling the progression of cancer [62]. Apart from Th9 cells, the cytokine *IL-9* has also been widely explored in cancers. Expression of *IL-9* in the serum and circulating CD4^+^ T cells was significantly upregulated in patients with breast cancer compared with healthy controls [63]. Purwar et al [10] demonstrated that *IL-9* depletion in *RORγt*-deficient mice promoted melanoma growth. Zheng et al [64] demonstrated that Th9 cells produce *IL-9* to induce glioma cell apoptosis and inhibit tumor growth. Interestingly, tumor-specific Th9 cells displayed a unique *PU.1-TRAF6-NF-kB* activation–driven hyperproliferative feature, suggesting a persistence mechanism rather than an antiapoptotic strategy. This equips tumor-specific Th9 cells to become a more effective CD4^+^ T-cell subset for adoptive cancer therapy [65]. Although Th9 cells play an important role in tumor suppression, they have not been studied in various cancer subtypes. Our analysis suggests a possible role for Th9 in different cancer types such as malignant neoplasm of the stomach, melanoma, neuroblastoma, osteosarcoma, pancreatic carcinoma, and prostate carcinoma. Finally, our study also highlights the possible role of Th9 in different metabolic diseases. Interestingly, to our knowledge, no study has yet explored the role of Th9 in metabolic diseases such as diabetes and obesity. We want to highlight these lacunae to open up newer research attempts that would explore the role of Th9 in metabolic diseases. The insights into the role of Th9 in metabolic diseases would better help delineate the role of immunological dysregulation in developing metabolic diseases.

**Table 1:** Role of various differentially expressed genes in the differentiation of T helper 9 cells and production of *IL-9*^a^.

Cytokine or ligand	Receptor	Transcription factors	Effect on T helper 9 cell differentiation	References
*IL-6*	*IL-6R* and *gp130*	*STAT1*^b^ and *STAT3*	Both increases and decreases	[66,67]
*IL-10*	*IL-10R1*^c^ and *IL-10R2*	*STAT1* and *STAT3*	Both increases and decreases	[68-70]
*IL-23*	*IL-23R* and *IL-12RB1*	*STAT3*	Decreases	[71,72]
*IL-27*	*IL-27R* and *gp130*	*STAT1*	Decreases	[73]
*IL-27*	*IL-27R*	*IFN-γ* ^d^	Decreases	[74]
*IL-1α*	*IL-1R1* and *IL-1RACP*	*NF-κB*^e^,* MYD88*^f^, and *IRAK*^g^	Increases	[75,76]
*IL-1β*	*IL-1R1* and *IL-1RACP*	*MYD88, IRAK, NF-κB, STAT1, IL-9,* and *IRF1*^h^	Increases	[77-80]
*IL-2*	*IL-2Rα, IL-2Rβ,* and *γc*	*STAT5, IL-9, BCL-6*^i^*, IRF4,* and *GATA3*^j^	Increases	[75,81,82]
*IL-4*	*IL-4Rα* and *γ-chain*	*STAT6, FOXP3^k^, IL-9*	Increases	[83-85]
*IL-21*	*IL-21R* and *γ-chain*	*IL-1β, BCL-6, STAT1,* and *STAT3*	Increases	[77,82]
*IL-25*	*IL-17RB*	*ACT1*^l^ and *TRAF6?*^m^	Increases	[86]
*IL-33*	*IL-1RL1* and *IL-1RACP*	Unknown	Increases	[87]
*IFNα* and *IFNβ*	*IFNAR1*^n^ and *IFNAR2*	*STAT1*	Increases	[69]
*TGFβ* ^o^	*TGFβR2*	*SMAD^p^, IL-9, PU.1^q^, FOXP3*	Increases	[85,88,89]
*TSLP* ^r^	*TSLPR*^s^ and *IL-7Rα*	*STAT5, IL-9*	Increases	[81]
*Activin A*	*ACTRII*^t^ and *ALK4*^u^	*SMAD, TGFβ*	Increases	[90]
*CGRP* ^v^	N/A^w^	*PKA*^x^*, NFATC2*^y^*, GATA3,* and *PU.1*	Increases	[91]
*Nitric oxide*	N/A	*p53^z^, IL-2, STAT5, IL-4Rα, TGFβR2*	Increases	[15]
*TL1A* ^aa^	*DR3* ^bb^	*IL-2, STAT5*	Increases	[92]
*Notch*	Jagged	*NICD1* ^cc^	Increases	[67]
*IFNγ*	*IFNGR1*^dd^ and *IFNGR2*^ee^	*STAT1*	Decreases	[73]
*PDL2* ^ff^	*PD1* ^gg^	*SHP2* ^hh^	Decreases	[93]

^a^IL: interleukin.

^b^*STAT*: signal transducer and activator of transcription.

^c^*ILxR*: interleukin receptor (where x corresponds to the interleukin number).

^d^IFN: interferon.

^e^*NF-κB*: nuclear factor kappa B.

^f^*MYD88*: myeloid differentiation primary response gene 88.

^g^*IRAK*: interleukin-1 receptor-associated kinase 1.

^h^*IRF*: interferon regulatory factor.

^i^BCL-6: B-cell leukemia/lymphoma 6.

^j^*GATA3*: GATA binding protein 3.

^k^*FOXP3*: forkhead box P3.

^l^*ACT1*: actin-related gene 1.

^m^*TRAF6*: TNF receptor–associated factor 6.

^n^*IFNAR*: interferon (alpha and beta) receptor.

^o^*TGF*: transforming growth factor.

^p^*SMAD*: SMAD family member.

^q^*PU.1*: spleen focus forming virus (SFFV) proviral integration oncogene.

^r^*TSLP*: thymic stromal lymphopoietin.

^s^*TSLPR*: thymic stromal lymphopoietin receptor.

^t^*ACTRII*: activin receptor type 2.

^u^*ALK4*: activin A receptor, type 1B.

^v^*CGRP*: calcitonin/calcitonin-related polypeptide.

^w^N/A: not applicable.

^x^*PKA*: protein kinase A.

^y^*NFATC2*: nuclear factor of activated T cells, cytoplasmic, calcineurin dependent 2.

^z^*p53*: transformation-related protein 53.

^aa^*TL1A*: tumor necrosis factor (ligand) superfamily, member 15.

^bb^*DR3*: death-domain receptor 3 (tumor necrosis factor receptor superfamily).

^cc^*NICD1*: notch1 intracellular domain 1.

^dd^*IFNGR1*: interferon gamma receptor 1.

^ee^*IFNGR2*: interferon gamma receptor 2.

^ff^*PDL2*: programmed cell death 1 ligand 2.

^gg^*PD1*: programmed cell death protein 1.

^hh^*SHP2*: protein tyrosine phosphatase, nonreceptor type 11.

**Table 2:** Role of various microRNAs and target genes in the differentiation of Th9^a^ cells in various disease conditions.

MicroRNA	Study model	Type of disease	Level of microRNA	Molecular target gene	Differentiation of Th9	Reference
miR-145	Mouse	Liver cancer	Upregulated	Reducing the expression of *HIF-1α*^b^	Increased	[94]
miR-155	Mouse	Wound	Upregulated	Increased *c-MAF1*^c^*, SOCS1*^d^*, CXCL1*^e^*, CXCL2*^f^*, IL-9R*^g^*/IL-9*^h^*, IL-17R*^i^*/IL-17A*	Increased	[95]
miR-155	Human and mouse	Acute graft-versus-host disease	Upregulated	*TNF-α* ^j^	Increased	[96]
miR-15b/miR-16	Mouse	N/A^k^	Upregulated	Decreased *HIF-2α* expression	Decreased the *IL-9* level in overexpressed Th9 cells	[97]
miR-493-5p	Both human and mouse	Asthma	Downregulated	Decreased *FOXO1*^l^ expression	Decreased	[98]
miR-143 and miR-145	Mouse	N/A	Upregulated	*NFATC1*^m^ downregulation	Decreased	[99]
miR-155	Human	Methicillin-resistant *Staphylococcus aureus* pneumonia	Upregulated	Decreased *SIRT1*^n^	Increased Th9/*IL-9*	[100]
miR-148a-3p	Mouse	Allergic rhinitis	Upregulated	Increased *IRF4*^o^	Increased	[101]

^a^Th9: T helper 9.

^b^*HIF*: hypoxia-inducible factor.

^c^*MAF*: avian musculoaponeurotic fibrosarcoma oncogene homolog.

^d^*SOCS1*: suppressor of cytokine signaling 1.

^e^*CXCL1*: chemokine (C-X-C motif) ligand 1.

^f^*CXCL2*: chemokine (C-X-C motif) ligand 2.

^g^*IL-9R*: interleukin 9 receptor.

^h^*IL*: interleukin.

^i^*IL-17R*: interleukin 17 receptor.

^j^*TNF*: tumor necrosis factor.

^k^N/A: not applicable.

^l^*FOXO1*: forkhead box O1.

^m^*NFATC1*: nuclear factor of activated T cells, cytoplasmic, calcineurin dependent 1.

^n^*SIRT1*: sirtuin 1.

^o^*IRF*: interferon regulatory factor.

### Limitations

The main limitation of the study is that the analysis is based on an in silico method where only a few specific wild-type samples from data sets of previous studies were included; therefore, further validation of the identified genes and miRNAs is required in various animal models and human diseases. The data sets were compiled using different arrays on the Affymetrix platform, which may account for some of the variability in the results. However, the functional enrichment for the mRNAs highlighted some significant pathways related to immune regulation and its derangements.

### Conclusions

This study identified common DEGs of ILs, receptors, and TFs that have significantly altered expression between Th2 and Th9 cells. The KEGG pathway enrichment analysis identified cytokines-cytokines interaction, Th1 and Th2 differentiation, T-cell receptor signaling regulation via CTLA4, Fc epsilon signaling, and Th17 cell differentiation as the significant pathways affected by the identified DEGs. Our study identified hitherto unexplored possible associations between Th9 and disease states. The interactome analysis also identified pathways that are involved in various metabolic diseases, allergic and pulmonary diseases, carcinomas, neuropsychiatric disorders, autoimmune diseases, and infectious diseases, where differentiation of Th2 to Th9 may play a crucial role. The scarcity of studies on the role of Th9 in metabolic diseases highlights the lacunae in this field. Thus, our study provides the rationale for exploring the role of Th9 in various metabolic disorders.

## Appendix

Multimedia Appendix 1 Fold change expression of the significant differentially expressed genes analyzed.

Multimedia Appendix 2 Higher resolution images for Figures 3-7.

## Abbreviations

*ACT1*: actin-related gene 1
*ACTRII*: activin receptor type 2
*AHR*: aryl-hydrocarbon receptor
*ALK4*: activin A receptor, type 1B
*ATF6*: activating transcription factor 6
*BAFT*: basic leucine zipper transcription factor
*BCL6*: B-cell leukemia/lymphoma 6
*BMP7*: bone morphogenetic protein 7
*BTAF1*: B-TFIID TATA-box binding protein associated factor 1
*CCL1*: chemokine (C-C motif) ligand 1
*CCL20*: chemokine (C-C motif) ligand 20
*CCR6*: chemokine (C-C motif) receptor 6
CD: cluster of differentiation
*CGRP*: calcitonin/calcitonin-related polypeptide, alpha
CTLA: cytotoxic T lymphocyte–associated protein
*CX3CR1*: chemokine (C-X3-C motif) receptor 1
*CXCL1*: chemokine (C-X-C motif) ligand 1
*CXCL2*: chemokine (C-X-C motif) ligand 2
DC: dendritic cell
DEG: differentially expressed gene
*DR3*: death-domain receptor 3 (tumor necrosis factor receptor superfamily)
EAE: autoimmune encephalomyelitis
*EP300*: E1A binding protein p300
*FOXO1*: forkhead box O1
*FOXP2*: forkhead box P2
*FOXP3*: forkhead box P3
*GATA3*: GATA binding protein 3
GEO: Gene Expression Omnibus
GO: The Gene Ontology
GREIN: GEO RNA-seq Experiments Interactive Navigator
*HIF*: hypoxia-inducible factor
IBD: inflammatory bowel disease
IF1: NDV-induced circulating interferon
IFN: interferon
*IFNAR1*: interferon (alpha and beta) receptor 1
*IFNAR2*: interferon (alpha and beta) receptor 2
*IFNGR1*: interferon gamma receptor 1
*IFNGR2*: interferon gamma receptor 2
IL: interleukin
*IL-1R1*: interleukin 1 receptor, type I
*IL-1RL1*: interleukin 1 receptor-like 1
*IL-1RL1*: interleukin 1 receptor-like 1
*IL-2R*: interleukin 2 receptor, alpha chain
*IL-4R*: interleukin 4 receptor, alpha
*IL-4RA*: interleukin 4 receptor, alpha
*IL-6R*: interleukin 6 receptor, alpha
*IL-7R*: interleukin 7 receptor
*IL-9R*: interleukin 9 receptor
*IL-10R2*: interleukin 10 receptor, beta
*IL-12RB1*: interleukin 12 receptor, beta 1
*IL-12RB1*: interleukin 12 receptor, beta 1
*IL-17R*: interleukin 17 receptor A
*IL-17RB*: interleukin 17 receptor B
*IL-21R*: interleukin 21 receptor
*IL-23R*: interleukin 23 receptor
IRAK: interleukin-1 receptor-associated kinase 1
*IRF1*: interferon regulatory factor
*JAK2*: Janus kinase 2
*JUN*: Jun proto-oncogene
KEGG: Kyoto Encyclopedia of Genes and Genomes
*MAF*: avian musculoaponeurotic fibrosarcoma oncogene homolog
*MAPK*: mitogen-activated protein kinase
MS: multiple sclerosis
*MYD88*: myeloid differentiation primary response gene 88
*NFATC1*: nuclear factor of activated T cells, cytoplasmic, calcineurin dependent 1
*NFATC2*: nuclear factor of activated T cells, cytoplasmic, calcineurin dependent 2
NF-κB: nuclear factor kappa B
*NICD1*: notch1 intracellular domain 1
*NOTCH1*: neurogenic locus notch homolog protein 1
*p53*: transformation-related protein 53
*PD1*: programmed cell death protein 1
*PDL2*: programmed cell death 1 ligand 2
*PPARG*: peroxisome proliferator–activated receptor gamma
PPI: protein-protein interaction
*PU.1*: spleen focus forming virus (SFFV) proviral integration oncogene
*R2*: ribonucleotide reductase M2
*RORA*: RAR-related orphan receptor alpha
*SATB1*: special AT-rich sequence binding protein 1
*SGK1*: serum/glucocorticoid regulated kinase 1
*SHP2*: protein tyrosine phosphatase, non-receptor type 11
*SIRT1*: sirtuin 1
SLE: systemic lupus erythematosus
*SMAD3*: SMAD family member 3
*SMAD4*: SMAD family member 4
*SMAD6*: SMAD family member 6
*SOCS1*: suppressor of cytokine signaling 1
*STAT*: signal transducer and activator of transcription
TGF: transforming growth factor
Th: T helper
*TL1A*: tumor necrosis factor (ligand) superfamily, member 15
TLR: Toll-like receptor
TNF: tumor necrosis factor
*TRAF6*: TNF receptor–associated factor 6
*TSLP*: thymic stromal lymphopoietin
*TSLPR*: thymic stromal lymphopoietin receptor

## Data Availability

Publicly available GEO data sets were used for the analysis in this study. These data sets can be accessed online [102,103].
