# Supplementary material for: The Differentially Expressed Genes Responsible for the Development of T Helper 9 Cells From T Helper 2 Cells in Various Disease States: Immuno-Interactomics Study
Source: JMIR Bioinform Biotechnol. 2023 Feb 23;4:e42421. doi: 10.2196/42421 (PMC11135241; doi:10.2196/42421)
Supplement: Multimedia Appendix 1 [file bioinform_v4i1e42421_app1.docx]

## Multimedia Appendix 1:

**Supplementary Table 1.** The Fold Change expression of significant Down Regulated DEGs Involved in Th2 to Th9 cells differentiation.

| Gene Symbol | FCE (GSE123501) | FCE (GSE99166) |
| --- | --- | --- |
| ABCB1B | 0.41 | 0.54 |
| ABCB9 | 0.40 | 0.61 |
| AEN | 0.40 | 0.70 |
| AFP | 0.46 | 0.48 |
| AGO2 | 0.37 | 0.64 |
| AI504432 | 0.13 | 0.35 |
| ANKRD13B | 0.33 | 0.68 |
| APOBEC1 | 0.48 | 0.48 |
| ARHGAP10 | 0.44 | 0.69 |
| ARID5B | 0.48 | 0.56 |
| ARMCX6 | 0.38 | 0.62 |
| ATF6 | 0.43 | 0.65 |
| ATM | 0.47 | 0.52 |
| ATXN1 | 0.30 | 0.59 |
| B3GNT2 | 0.28 | 0.71 |
| BATF | 0.36 | 0.58 |
| BCL2L11 | 0.46 | 0.66 |
| BCL7A | 0.46 | 0.72 |
| BMP7 | 0.21 | 0.34 |
| BSPRY | 0.43 | 0.34 |
| BTAF1 | 0.44 | 0.65 |
| BTLA | 0.32 | 0.67 |
| CABIN1 | 0.38 | 0.75 |
| CALCRL | 0.11 | 0.49 |
| CAR12 | 0.57 | 0.67 |
| CASP1 | 0.14 | 0.53 |
| CASP4 | 0.19 | 0.46 |
| CASP6 | 0.39 | 0.47 |
| CBLB | 0.45 | 0.62 |
| CCDC15 | 0.36 | 0.37 |
| CD5 | 0.19 | 0.75 |
| CD53 | 0.19 | 0.60 |
| CD6 | 0.21 | 0.69 |
| CDK12 | 0.47 | 0.73 |
| CDK6 | 0.39 | 0.76 |
| CELSR1 | 0.21 | 0.54 |
| CHD2 | 0.44 | 0.65 |
| CHST15 | 0.38 | 0.63 |
| CLUH | 0.33 | 0.63 |
| CNNM1 | 0.40 | 0.32 |
| COX15 | 0.46 | 0.74 |
| CSF2 | 0.18 | 0.35 |
| CST7 | 0.11 | 0.55 |
| CYFIP2 | 0.34 | 0.70 |
| D16ERTD472E | 0.40 | 0.72 |
| D930015E06RIK | 0.42 | 0.57 |
| DAB2IP | 0.36 | 0.36 |
| DAPK1 | 0.41 | 0.60 |
| DENND2D | 0.41 | 0.72 |
| DHX37 | 0.35 | 0.72 |
| DNA2 | 0.41 | 0.65 |
| DOCK5 | 0.38 | 0.54 |
| DROSHA | 0.37 | 0.63 |
| DUSP6 | 0.34 | 0.71 |
| EEA1 | 0.45 | 0.63 |
| EHD2 | 0.43 | 0.18 |
| EHD4 | 0.36 | 0.62 |
| EP300 | 0.43 | 0.71 |
| EPAS1 | 0.08 | 0.35 |
| ETV6 | 0.32 | 0.54 |
| EVL | 0.19 | 0.75 |
| F2RL2 | 0.30 | 0.26 |
| FAM71B | 0.31 | 0.44 |
| FEM1A | 0.43 | 0.72 |
| FERMT3 | 0.38 | 0.74 |
| FILIP1L | 0.17 | 0.40 |
| FOCAD | 0.49 | 0.72 |
| FOSL1 | 0.15 | 0.71 |
| FRMD6 | 0.39 | 0.74 |
| GART | 0.37 | 0.73 |
| GATA3 | 0.23 | 0.55 |
| GBP2 | 0.29 | 0.53 |
| GBP9 | 0.01 | 0.47 |
| GLIPR2 | 0.50 | 0.62 |
| GM14718 | 0.37 | 0.59 |
| GM684 | 0.16 | 0.31 |
| GPM6B | 0.35 | 0.46 |
| GPR183 | 0.31 | 0.52 |
| GRAMD1B | 0.18 | 0.76 |
| GRAP | 0.51 | 0.73 |
| GSR | 0.34 | 0.72 |
| GUSB | 0.39 | 0.63 |
| GYPC | 0.45 | 0.71 |
| HEATR1 | 0.32 | 0.65 |
| HMGA1 | 0.38 | 0.64 |
| HSPA1A | 0.42 | 0.58 |
| IARS | 0.41 | 0.74 |
| ICAM5 | 0.27 | 0.34 |
| IKZF3 | 0.23 | 0.72 |
| IL12RB1 | 0.23 | 0.35 |
| IL13 | 0.16 | 0.32 |
| IL4 | 0.13 | 0.36 |
| IL4RA | 0.26 | 0.60 |
| IQGAP2 | 0.51 | 0.62 |
| ITK | 0.39 | 0.70 |
| JAK2 | 0.27 | 0.75 |
| KAT6A | 0.39 | 0.70 |
| KBTBD11 | 0.39 | 0.57 |
| KCNA3 | 0.08 | 0.18 |
| KCNQ5 | 0.45 | 0.57 |
| KDELC2 | 0.47 | 0.67 |
| KDM2B | 0.39 | 0.69 |
| KSR1 | 0.18 | 0.66 |
| LARS2 | 0.46 | 0.64 |
| LCP2 | 0.30 | 0.75 |
| LIG3 | 0.47 | 0.72 |
| LILR4B | 0.14 | 0.19 |
| LRMP | 0.40 | 0.69 |
| LY6A | 0.13 | 0.16 |
| MAN1A | 0.34 | 0.77 |
| MAPK12 | 0.48 | 0.25 |
| MAPRE2 | 0.34 | 0.57 |
| MDN1 | 0.35 | 0.57 |
| MLLT3 | 0.46 | 0.73 |
| MS4A6B | 0.44 | 0.76 |
| MTHFD1 | 0.39 | 0.74 |
| MTR | 0.37 | 0.67 |
| MTRR | 0.43 | 0.72 |
| MYBBP1A | 0.32 | 0.63 |
| MYCBP2 | 0.47 | 0.72 |
| MYO18A | 0.40 | 0.61 |
| MYO9B | 0.42 | 0.63 |
| NCOA7 | 0.49 | 0.69 |
| NCOR2 | 0.29 | 0.65 |
| NEFH | 0.42 | 0.63 |
| NFIX | 0.34 | 0.49 |
| NKD2 | 0.40 | 0.44 |
| NME6 | 0.25 | 0.59 |
| NOL9 | 0.36 | 0.74 |
| NOTCH1 | 0.31 | 0.57 |
| NUP188 | 0.33 | 0.72 |
| NUP205 | 0.44 | 0.60 |
| OAS3 | 0.26 | 0.33 |
| P2RY1 | 0.16 | 0.48 |
| PDCD11 | 0.30 | 0.72 |
| PFAS | 0.34 | 0.67 |
| PHF11B | 0.25 | 0.46 |
| PITPNM2 | 0.37 | 0.74 |
| PLXNA1 | 0.37 | 0.58 |
| PLXNB2 | 0.28 | 0.34 |
| PLXNC1 | 0.24 | 0.43 |
| PLXND1 | 0.33 | 0.49 |
| POLR1A | 0.46 | 0.41 |
| POLR1B | 0.36 | 0.64 |
| POLR3B | 0.44 | 0.67 |
| POP1 | 0.42 | 0.70 |
| POU2AF1 | 0.31 | 0.65 |
| PPARG | 0.21 | 0.27 |
| PPIP5K2 | 0.49 | 0.74 |
| PPP1R10 | 0.37 | 0.73 |
| PPRC1 | 0.33 | 0.72 |
| PREP | 0.37 | 0.76 |
| PRPF8 | 0.35 | 0.69 |
| PRRC2C | 0.40 | 0.73 |
| PTK7 | 0.32 | 0.37 |
| PTPN5 | 0.07 | 0.29 |
| PTPN7 | 0.43 | 0.76 |
| RAB19 | 0.43 | 0.54 |
| RAB43 | 0.32 | 0.73 |
| RASAL1 | 0.36 | 0.58 |
| RERE | 0.28 | 0.61 |
| RNF213 | 0.24 | 0.48 |
| RPUSD2 | 0.47 | 0.69 |
| RREB1 | 0.46 | 0.67 |
| RRP12 | 0.38 | 0.70 |
| SATB1 | 0.12 | 0.53 |
| SCAF1 | 0.33 | 0.67 |
| SDC1 | 0.54 | 0.48 |
| SEC16A | 0.44 | 0.76 |
| SERPINB9 | 0.50 | 0.36 |
| SERPINF1 | 0.45 | 0.37 |
| SESN2 | 0.29 | 0.67 |
| SETD2 | 0.44 | 0.53 |
| SF3B3 | 0.40 | 0.72 |
| SHMT1 | 0.37 | 0.68 |
| SHTN1 | 0.17 | 0.44 |
| SIK1 | 0.29 | 0.66 |
| SIK3 | 0.25 | 0.60 |
| SIPA1L1 | 0.31 | 0.58 |
| SLAMF1 | 0.50 | 0.64 |
| SLAMF7 | 0.29 | 0.31 |
| SLC35F2 | 0.35 | 0.68 |
| SLC38A1 | 0.37 | 0.76 |
| SLC39A14 | 0.55 | 0.75 |
| SLFN10-PS | 0.47 | 0.58 |
| SMO | 0.41 | 0.69 |
| SMYD5 | 0.38 | 0.65 |
| SNAPC4 | 0.41 | 0.68 |
| SNX19 | 0.46 | 0.72 |
| SNX9 | 0.14 | 0.48 |
| SP6 | 0.29 | 0.52 |
| SRGN | 0.42 | 0.64 |
| SRRT | 0.23 | 0.34 |
| ST3GAL5 | 0.50 | 0.61 |
| STAP1 | 0.52 | 0.56 |
| TANC2 | 0.26 | 0.39 |
| TATDN2 | 0.49 | 0.70 |
| TBRG4 | 0.42 | 0.76 |
| TCF20 | 0.45 | 0.70 |
| TCOF1 | 0.27 | 0.75 |
| TEX15 | 0.32 | 0.48 |
| TIAM1 | 0.28 | 0.64 |
| TIGIT | 0.24 | 0.60 |
| TMEM229B | 0.23 | 0.59 |
| TNFRSF8 | 0.14 | 0.26 |
| TNFSF8 | 0.27 | 0.41 |
| TNRC18 | 0.36 | 0.70 |
| TRIM44 | 0.48 | 0.75 |
| TRP53BP1 | 0.51 | 0.74 |
| TSPAN4 | 0.40 | 0.39 |
| UBR4 | 0.37 | 0.66 |
| UGT3A2 | 0.51 | 0.20 |
| UPF1 | 0.30 | 0.73 |
| UPRT | 0.51 | 0.62 |
| URB1 | 0.33 | 0.68 |
| URB2 | 0.40 | 0.66 |
| USO1 | 0.44 | 0.66 |
| USP36 | 0.38 | 0.74 |
| UTP20 | 0.35 | 0.58 |
| VAMP5 | 0.16 | 0.45 |
| VAV3 | 0.53 | 0.68 |
| VPS13A | 0.48 | 0.71 |
| WDR26 | 0.42 | 0.76 |
| WNT10A | 0.31 | 0.31 |
| XPO4 | 0.46 | 0.63 |
| ZC3H4 | 0.38 | 0.67 |
| ZC3H7B | 0.34 | 0.58 |
| ZFP507 | 0.52 | 0.54 |
| ZFP780B | 0.00 | 0.13 |
| ZMAT3 | 0.37 | 0.73 |

**Supplementary Table 2.** The Fold Change expression of significant Up-regulated DEGs Involved in Th2 to Th9 cells differentiation.

| Gene Symbol | FCE (GSE99166) | FCE (GSE123501) |
| --- | --- | --- |
| ACSBG1 | 6.37 | 14.48 |
| ACTR3B | 2.28 | 4.72 |
| ACVR1C | 4.28 | 4.08 |
| ADAM19 | 9.04 | 32.13 |
| ADAMTS6 | 2.06 | 1.91 |
| ADH1 | 3.24 | 3.72 |
| AHR | 6.73 | 4.74 |
| AIM2 | 1.61 | 2.45 |
| AKAP14 | 15.67 | 3.32 |
| ALOX5AP | 2.87 | 4.16 |
| ANKRD55 | 4.82 | 2.68 |
| ANXA4 | 1.71 | 2.08 |
| APOL9A | 46.46 | 3.91 |
| APOL9B | 484.05 | 30.34 |
| AQP3 | 36.53 | 14.23 |
| ARHGAP24 | 4.52 | 3.62 |
| ART2A-PS | 3.14 | 4.37 |
| ASB10 | 18.09 | 2.87 |
| ATP1A2 | 13.46 | 3.99 |
| ATP1B1 | 4.00 | 2.64 |
| ATRAID | 2.03 | 1.80 |
| AUTS2 | 2.19 | 2.46 |
| AVPR1A | 34.23 | 39.42 |
| B130006D01RIK | 2.07 | 2.39 |
| B4GALT4 | 3.34 | 2.56 |
| BAMBI | 1.86 | 2.43 |
| BEND5 | 1.60 | 6.25 |
| BEND6 | 2.22 | 2.79 |
| BMYC | 1.81 | 2.60 |
| C230024C17RIK | 3.79 | 2.43 |
| CACNA1D | 2.32 | 2.10 |
| CACNA1G | 3.26 | 3.81 |
| CACNB2 | 2.30 | 2.41 |
| CADM1 | 2.99 | 3.64 |
| CALN1 | 2.81 | 2.38 |
| CAMK2N1 | 4.20 | 2.05 |
| CAPG | 3.36 | 2.57 |
| CAR8 | 3.53 | 3.61 |
| CBR3 | 15.26 | 3.24 |
| CC2D2A | 5.92 | 3.25 |
| CCL1 | 13.86 | 2.12 |
| CCL20 | 27.27 | 6.50 |
| CCR6 | 4.33 | 26.96 |
| CCR7 | 1.82 | 2.19 |
| CD101 | 2.27 | 4.26 |
| CD38 | 2.11 | 7.30 |
| CD5L | 6.22 | 2.88 |
| CD7 | 4.30 | 3.43 |
| CD72 | 4.28 | 4.30 |
| CD83 | 1.57 | 2.09 |
| CD96 | 2.36 | 3.81 |
| CDH1 | 12.01 | 2.91 |
| CECR6 | 6.84 | 2.78 |
| CELF4 | 1.98 | 2.94 |
| CHIL3 | 2.84 | 4.29 |
| CIB2 | 3.57 | 5.59 |
| COL4A3 | 3.90 | 2.60 |
| COX17 | 1.39 | 2.50 |
| COX7A1 | 2.58 | 5.08 |
| CREBRF | 1.51 | 1.99 |
| CRIP1 | 1.70 | 2.57 |
| CRMP1 | 2.27 | 4.66 |
| CTLA2A | 5.90 | 10.50 |
| CTLA2B | 18.97 | 20.52 |
| CTLA4 | 6.93 | 3.08 |
| CTSW | 17.40 | 66.44 |
| CX3CR1 | 1.96 | 9.63 |
| CYP2D22 | 1.69 | 2.79 |
| CYP39A1 | 2.61 | 2.43 |
| DAPK2 | 4.00 | 3.49 |
| DKK3 | 8.19 | 3.02 |
| DMGDH | 3.25 | 3.02 |
| DSP | 4.26 | 2.07 |
| DST | 2.50 | 1.88 |
| DUSP14 | 1.72 | 3.10 |
| EMP1 | 10.92 | 15.83 |
| ENDOD1 | 5.21 | 3.29 |
| ENO3 | 1.48 | 2.10 |
| EPB41L5 | 1.62 | 2.43 |
| ERG | 2.85 | 2.41 |
| F2RL1 | 1.82 | 4.82 |
| FAM20A | 4.79 | 2.51 |
| FAM46A | 1.65 | 2.27 |
| FBLN2 | 3.96 | 4.15 |
| FBXO32 | 1.95 | 3.92 |
| FCHSD2 | 1.84 | 1.99 |
| FCRL1 | 14.35 | 3.50 |
| FES | 5.29 | 36.33 |
| FGGY | 2.05 | 2.20 |
| FGL2 | 5.23 | 2.89 |
| FHAD1 | 3.92 | 2.36 |
| FNBP1L | 1.72 | 2.28 |
| FOXP2 | 3.22 | 3.59 |
| FOXP3 | 4.45 | 23.36 |
| FRAT2 | 1.69 | 1.94 |
| FRMPD1 | 9.85 | 2.74 |
| FSD2 | 1.59 | 2.40 |
| GABRR2 | 1.83 | 2.81 |
| GALM | 2.26 | 2.35 |
| GALNT9 | 2.77 | 5.57 |
| GATM | 2.25 | 5.94 |
| GM13546 | 3.14 | 29.65 |
| GM30292 | 5.55 | 2.11 |
| GM527 | 2.26 | 2.72 |
| GOLM1 | 1.37 | 2.58 |
| GPR160 | 1.87 | 2.91 |
| GPR25 | 5.60 | 13.64 |
| GPR65 | 1.94 | 2.29 |
| GPRC5A | 1.89 | 2.27 |
| GSAP | 2.26 | 3.31 |
| GSTA4 | 8.78 | 9.65 |
| HIC1 | 1.71 | 3.73 |
| HRC | 2.73 | 4.87 |
| HSPB11 | 1.43 | 1.98 |
| IFI203 | 1.95 | 2.85 |
| IFIT1BL1 | 3.39 | 3.13 |
| IFIT3 | 2.53 | 2.93 |
| IFIT3B | 10.38 | 10.37 |
| IGFLR1 | 2.32 | 6.21 |
| IGSF11 | 3.10 | 2.64 |
| IL12A | 4.61 | 24.30 |
| IL1RL1 | 2.43 | 2.39 |
| IL9 | 4.93 | 12.69 |
| IRF8 | 5.19 | 10.37 |
| ITGAE | 10.07 | 1.98 |
| JUN | 1.54 | 2.47 |
| KCNF1 | 5.12 | 2.83 |
| KCNIP2 | 5.12 | 2.98 |
| KCTD14 | 7.94 | 5.07 |
| KLHL2 | 1.40 | 32.47 |
| KLRA7 | 2.34 | 12.34 |
| KLRD1 | 3.61 | 3.58 |
| KRT83 | 15.37 | 21.39 |
| LCN10 | 21.78 | 3.46 |
| LDLRAD4 | 3.74 | 4.80 |
| LGALS3 | 3.78 | 5.38 |
| LHX6 | 3.64 | 3.21 |
| LINGO4 | 3.38 | 2.96 |
| LRRC46 | 2.81 | 3.83 |
| LTA | 2.90 | 3.08 |
| LTB | 1.56 | 2.16 |
| LY6D | 2.16 | 6.57 |
| MAF | 7.63 | 3.88 |
| MAGED1 | 1.96 | 3.28 |
| MAGED2 | 2.32 | 2.07 |
| ME2 | 1.48 | 2.08 |
| MGAT5 | 2.42 | 3.18 |
| MGST3 | 3.18 | 3.73 |
| MN1 | 2.57 | 2.54 |
| MPZL1 | 3.21 | 2.06 |
| MPZL2 | 2.56 | 3.75 |
| MTURN | 1.63 | 2.26 |
| MXD4 | 1.81 | 2.00 |
| MYO3B | 6.04 | 4.24 |
| MYOF | 2.32 | 4.62 |
| MYT1 | 10.33 | 3.32 |
| NAV1 | 2.28 | 3.42 |
| NCMAP | 4.57 | 2.21 |
| NEBL | 2.03 | 2.83 |
| NEURL3 | 3.69 | 6.16 |
| NIPAL1 | 3.34 | 6.48 |
| NMRK1 | 1.63 | 2.62 |
| NPPC | 5.66 | 6.52 |
| NT5E | 13.70 | 20.03 |
| NTF5 | 2.29 | 2.90 |
| NTN5 | 20.84 | 2.65 |
| ODF3B | 2.38 | 3.42 |
| OSTF1 | 1.60 | 2.78 |
| OXT | 2.54 | 4.46 |
| PARVB | 2.66 | 2.76 |
| PDE4A | 4.09 | 1.85 |
| PDGFRL | 2.38 | 4.80 |
| PGPEP1L | 9.42 | 8.47 |
| PHACTR1 | 3.38 | 2.19 |
| PHLDA1 | 5.86 | 3.11 |
| PHLDB2 | 5.51 | 3.82 |
| PIGR | 3.58 | 5.03 |
| PIK3R6 | 10.20 | 2.73 |
| PLA1A | 12.03 | 6.36 |
| PLA2G4F | 4.28 | 5.42 |
| PLCB1 | 5.53 | 2.20 |
| PLPPR4 | 2.15 | 2.88 |
| PLXNA4 | 2.74 | 2.14 |
| POU6F2 | 2.22 | 2.99 |
| PRNP | 3.24 | 2.23 |
| PROCR | 4.54 | 2.21 |
| PRR5L | 4.48 | 2.65 |
| PTPN22 | 1.61 | 2.17 |
| QRFP | 2.57 | 2.48 |
| RAB11FIP4OS1 | 2.78 | 3.14 |
| RAB34 | 2.06 | 1.88 |
| RABAC1 | 1.37 | 2.48 |
| RAMP1 | 2.28 | 2.42 |
| RBM11 | 2.42 | 4.79 |
| RGS1 | 4.24 | 5.24 |
| RHOX8 | 1.88 | 4.24 |
| RORA | 4.34 | 4.76 |
| RPS27 | 2.31 | 5.63 |
| RRAS | 1.82 | 2.13 |
| RUNX1T1 | 2.71 | 2.81 |
| S100G | 4.89 | 4.75 |
| S1PR1 | 2.57 | 3.45 |
| SARDH | 9.30 | 4.07 |
| SCN1A | 3.16 | 2.84 |
| SEC1 | 15.50 | 3.31 |
| SERPINC1 | 1.71 | 3.31 |
| SERTM1 | 2.79 | 2.55 |
| SESTD1 | 1.86 | 2.31 |
| SFRP2 | 7.84 | 5.06 |
| SGK1 | 2.26 | 21.95 |
| SHANK1 | 2.38 | 4.56 |
| SHE | 1.96 | 3.93 |
| SIGLECH | 2.47 | 4.15 |
| SLC1A2 | 5.14 | 2.99 |
| SLC22A2 | 36.18 | 2.73 |
| SLITRK2 | 4.36 | 2.11 |
| SMAD6 | 10.04 | 3.22 |
| SMOX | 1.53 | 1.91 |
| SMPDL3A | 1.90 | 3.87 |
| SNORD22 | 16.67 | 88.95 |
| SP8 | 3.13 | 3.52 |
| SPHK1 | 3.80 | 2.94 |
| SQRDL | 1.61 | 2.52 |
| STAB1 | 3.83 | 2.52 |
| SUSD3 | 1.82 | 2.86 |
| SYP | 8.37 | 2.24 |
| SYT11 | 1.40 | 2.12 |
| SYT13 | 2.74 | 2.75 |
| TAX1BP3 | 1.54 | 2.15 |
| TBC1D4 | 4.43 | 3.43 |
| THBS1 | 3.81 | 4.16 |
| TLE2 | 2.71 | 2.25 |
| TLR1 | 3.26 | 2.73 |
| TMCO5B | 12.86 | 2.78 |
| TMEFF1 | 4.09 | 5.42 |
| TMEM151B | 2.81 | 2.63 |
| TMEM25 | 2.95 | 3.06 |
| TMEM65 | 1.74 | 1.93 |
| TNFRSF25 | 1.97 | 2.29 |
| TNFSF10 | 2.83 | 2.16 |
| TNNI1 | 2.04 | 5.77 |
| TRIM36 | 1.80 | 3.09 |
| TRIM43A | 20.28 | 5.86 |
| TTC39C | 2.13 | 5.14 |
| UNC93A | 2.47 | 2.59 |
| USP17LC | 3.28 | 5.39 |
| VMN2R97 | 3.24 | 3.86 |
| WDR86 | 6.66 | 2.66 |
| ZC2HC1A | 2.13 | 2.23 |
| ZFHX3 | 2.14 | 3.87 |
| ZFP945 | 1.74 | 2.77 |
| ZFP974 | 1.79 | 2.08 |

**Supplementary Table 3.** The Fold Change expression of significant DEGs of Immune Receptor Involved in Th2 to Th9 cells differentiation.

| Gene Symbol | FCE (GSE99166) | FCE (GSE123501) |
| --- | --- | --- |
| CD101 | 2.27 | 4.26 |
| CD38 | 2.11 | 7.30 |
| CD7 | 4.30 | 3.43 |
| CD72 | 4.28 | 4.30 |
| CD83 | 1.57 | 2.09 |
| CD96 | 2.36 | 3.81 |
| CD152 | 6.93 | 3.08 |
| CD196 | 4.33 | 26.96 |
| CD197 | 1.82 | 2.19 |
| CTLA2A | 5.90 | 10.50 |
| CTLA2B | 18.97 | 20.52 |
| CX3CR1 | 1.96 | 9.63 |
| IL1RL1 | 2.43 | 2.39 |
| CD5 | 0.75 | 0.19 |
| CD53 | 0.60 | 0.19 |
| CD6 | 0.69 | 0.21 |
| IL12RB1 | 0.35 | 0.23 |
| IL4RA | 0.60 | 0.26 |

**Supplementary Table 4.** The Fold Change expression of significant DEGs of Immune Cytokine/Chemokines Involved in Th2 to Th9 cells differentiation.

| Gene Symbol | FCE (GSE99166) | FCE (GSE123501) |
| --- | --- | --- |
| IL12A | 4.61 | 24.30 |
| CCL1 | 13.86 | 2.12 |
| CCL20 | 27.27 | 6.50 |
| IL9 | 4.93 | 12.69 |
| IL13 | 0.32 | 0.16 |
| IL4 | 0.36 | 0.13 |

**Supplementary Table 5.** The Fold Change expression of significant DEGs of Immune Transcription Factors Involved in Th2 to Th9 cells differentiation.

| Gene Symbol | FCE (GSE99166) | FCE (GSE123501) |
| --- | --- | --- |
| AHR | 6.73 | 4.74 |
| MAF | 7.63 | 3.88 |
| SMAD6 | 10.04 | 3.22 |
| IRF8 | 5.19 | 10.37 |
| FOXP2 | 3.22 | 3.59 |
| FOXP3 | 4.45 | 23.36 |
| RORA | 4.34 | 4.76 |
| NOTCH1 | 0.57 | 0.31 |
| BATF | 0.58 | 0.36 |
| GATA3 | 0.55 | 0.23 |
| SATB1 | 0.53 | 0.12 |
| ATF6 | 0.65 | 0.43 |
| BTAF1 | 0.65 | 0.44 |
| EP300 | 0.71 | 0.43 |
| PPARG | 0.27 | 0.21 |
| BMP7 | 0.34 | 0.21 |
| JAK2 | 0.75 | 0.27 |
| JUN | 2.47 | 1.54 |

**Supplementary Table 6.** Biological Process of common DEGs of interleukins, Receptors and Transcription Factors that co-differentially regulated in differentiation of Th2 to Th9 cells.

| KEGG Pathways | Observed gene count | Background gene count | Strength | False discovery rate | Protein Receptor |
| --- | --- | --- | --- | --- | --- |
| immune response | 53 | 1560 | 0.46 | 2.01E-09 | TNFSF8, CCL1, LRP1, CCR7, LGALS3, TLR2, THBS1, RORA, SEMA7A, LY75, TNFRSF8, TXK, IL21, RHOF, PFKL, IQGAP2, NOTCH1, CSF1R, SMAD6, NCF1, KPNB1, CSTB, GEM, CTLA4, RAB43, CD6, RORC, CCR4, BTLA, PTK2, SLPI, CTSL, LRMP, PIGR, ARG1, PAX5, DHX9, IL6R, S100A8, CTSS, PRDM1, PLAU, FGR, FOXP3, TNFRSF1B, GPR183, TNFRSF25, SIRPA, C4B, SEMA4D, CXCL2, ADAM8, CCL4 |
| inflammatory response | 26 | 482 | 0.66 | 8.81E-08 | CCL1, LRP1, CCR7, TLR2, THBS1, SEMA7A, LY75, TNFRSF8, NOTCH1, CSF1R, CD6, CCR4, FN1, PTGS2, DHX9, IL6R, S100A8, TSPAN2, THEMIS2, HSPG2, TNFRSF1B, TNFRSF25, C4B, CXCL2, ADAM8, CCL4 |
| positive regulation of cytokine production | 21 | 390 | 0.66 | 1.85E-06 | CCR7, TLR2, THBS1, RORA, SEMA7A, TNFRSF8, TXK, IL21, CSF1R, STAT5B, CD6, MYB, PTPN22, PTGS2, DHX9, IL6R, FGR, FOXP3, TIGIT, ZBTB20, ADAM8 |
| cell surface receptor signaling pathway | 55 | 2198 | 0.33 | 5.75E-06 | AGO2, TNFSF8, CCL1, LNPEP, SOX4, CCR7, TLR2, RORA, SEMA7A, CELSR1, TNFRSF8, CBLB, TXK, IL21, F13A1, NOTCH1, CSF1R, TIAM1, SMAD6, NCF1, STAT5B, GEM, CTLA4, S1PR1, ELMO1, CD6, RORC, CCR4, PTK2, STAT5A, FN1, PIGR, PTPN22, PTGS2, IL6R, TSPAN2, THEMIS2, FGR, SPEN, FOXP3, TNFRSF1B, TNFRSF25, FOXO1, AFP, GRB10, CTNND1, CALCRL, LDB1, PLXNB2, SEMA4D, CXCL2, CYFIP2, MYO9B, CCL4, CD24 |
| Defence response | 36 | 1234 | 0.39 | 3.59E-05 | TNFSF8, CCL1, LRP1, CCR7, LGALS3, TLR2, THBS1, SEMA7A, LY75, TNFRSF8, NOTCH1, CSF1R, NCF1, RAB43, CD6, CCR4, PTK2, SLPI, FN1, ARG1, PTGS2, DHX9, IL6R, S100A8, PRDM1, TSPAN2, THEMIS2, FGR, HSPG2, TNFRSF1B, TNFRSF25, C4B, EIF4G1, CXCL2, ADAM8, CCL4 |
| cellular response to cytokine stimulus | 30 | 953 | 0.43 | 6.09E-05 | TNFSF8, CCL1, CCR7, TLR2, THBS1, RORA, TNFRSF8, IL21, F13A1, CSF1R, HK2, STAT5B, S1PR1, RAB43, RORC, CCR4, STAT5A, DPYSL3, FN1, ARG1, PTGS2, DHX9, IL6R, TNFRSF1B, TNFRSF25, FOXO1, PFKP, CXCL2, PRPF8, CCL4 |
| cytokine-mediated signaling pathway | 21 | 655 | 0.43 | 0.001 | TNFSF8, CCL1, CCR7, RORA, TNFRSF8, IL21, F13A1, CSF1R, STAT5B, S1PR1, RORC, CCR4, STAT5A, FN1, PTGS2, IL6R, TNFRSF1B, TNFRSF25, FOXO1, CXCL2, CCL4 |

**Supplementary Table 7.** Cellular Components of common DEGs of interleukins, Receptors and Transcription Factors that co-differentially regulated in differentiation of Th2 to Th9 cells.

| Term description | observed gene count | background gene count | strength | Negative Log10 (FDR) | false discovery rate | matching proteins in your network (labels) |
| --- | --- | --- | --- | --- | --- | --- |
| Plasma membrane | 13 | 4328 | 0.46 | 2.2 | 0.007 | NCF1, CSF1R, IL6RA, TLR2, RORC, TNFRSF1B, TNFRSF8, FGR, CCR4, ELMO1, CCR7, PTK2, TIAM1 |
| Cell surface | 6 | 796 | 0.86 | 2.2 | 0.007 | CSF1R, IL6RA, TLR2, RORC, CCR4, CCR7 |
| Somatodendritic compartment | 6 | 990 | 0.76 | 2.2 | 0.007 | NCF1, IL6RA, TNFRSF1B, CCR4, PTK2, TIAM1 |
| Cell body | 6 | 776 | 0.87 | 2.2 | 0.007 | NCF1, IL6RA, TLR2, TNFRSF1B, CCR4, TIAM1 |
| Extracellular space | 6 | 1131 | 0.71 | 2.0 | 0.0098 | CCL4, IL21, IL6RA, TNFSF8, CXCL2, CCL1 |
| Cell-cell contact zone | 2 | 71 | 1.43 | 1.6 | 0.0244 | PTK2, TIAM1 |

**Supplementary Table 8.** Molecular Function of common DEGs of interleukins, Receptors and Transcription Factors that co-differentially regulated in differentiation of Th2 to Th9 cells.

| Term Description | observed gene count | background gene count | strength | Negative FDR | false discovery rate | matching proteins in your network (labels) |  |
| --- | --- | --- | --- | --- | --- | --- | --- |
| Signaling Receptor Binding | 11 | 1515 | 0.84 | 5.03 | 9.27E-06 | CCL4, IL21, IL6RA, TLR2, TNFSF8, FGR, CXCL2, STAT5B, CCL1, PTK2, TIAM1 | |
| Cytokine Receptor Binding | 6 | 267 | 1.33 | 4.77 | 1.71E-05 | CCL4, IL21, IL6RA, TNFSF8, CXCL2, CCL1 | |
| Signaling Receptor Activity | 9 | 1020 | 0.93 | 4.69 | 2.03E-05 | CSF1R, IL6RA, TLR2, RORC, TNFRSF1B, TNFRSF8, RORA, CCR4, CCR7 | |
| Cytokine Activity | 5 | 197 | 1.39 | 4.27 | 5.36E-05 | CCL4, IL21, TNFSF8, CXCL2, CCL1 | |
| Oxysterol Binding | 2 | 3 | 2.81 | 3.57 | 0.00027 | RORC, RORA | |
| Chemokine Activity | 3 | 41 | 1.85 | 3.55 | 0.00028 | CCL4, CXCL2, CCL1 | |
| Cytokine Receptor Activity | 3 | 88 | 1.51 | 2.80 | 0.0016 | IL6RA, CCR4, CCR7 | |

**Supplementary Table 9.** Seven most significant KEGG Pathways of common interatomically DEGs of interleukins, Receptors and Transcription Factors that co-differentially regulated in differentiation of Th2 to Th9 cells.

|  | number of nodes | number of edges | average node degree | avg. local clustering coefficient | expected number of edges | PPI enrichment p-value |
| --- | --- | --- | --- | --- | --- | --- |
| Pathways in cancer | 540 | 20522 | 76 | 0.587 | 7444 | < 1.0e-16 |
| T cell receptor signaling pathway | 103 | 2374 | 46.1 | 0.686 | 605 | < 1.0e-16 |
| Th1 and Th2 cell differentiation | 88 | 951 | 21.6 | 0.673 | 116 | < 1.0e-16 |
| Th17 cell differentiation | 105 | 1402 | 26.7 | 0.672 | 210 | < 1.0e-16 |
| Fc epsilon RI signaling pathway | 66 | 1110 | 33.6 | 0.743 | 271 | < 1.0e-16 |
| Inflammatory bowel disease (IBD) | 61 | 798 | 26.2 | 0.82 | 83 | < 1.0e-16 |
| Th1 and Th2 cell differentiation | 293 | 7551 | 51.5 | 0.601 | 647 | < 1.0e-16 |

**Supplementary Table 10.** KEGG Pathways of common DEGs of interleukins, Receptors and Transcription Factors that co-differentially regulated in differentiation of Th2 to Th9 cells.

| KEGG Pathways | observed gene count | background gene count | strength | false discovery rate | matching proteins in your network (labels) |
| --- | --- | --- | --- | --- | --- |
| Cytokine-cytokine receptor interaction | 19 | 252 | 0.55 | 0.0013 | Il12rb1, Il4, Bmp7, Csf2, Il13, Il9, Ltb, Lta, Il12a, Tnfsf8, Tnfrsf8, Il4ra, Tnfsf10, Tnfrsf25, Cx3cr1, Ccr6, Ccr7, Ccl1, Ccl20 |
| Th1 and Th2 cell differentiation | 11 | 86 | 0.78 | 0.0013 | Il12rb1, Il4, Il13, Notch1, Il12a, Il4ra, Jak2, Mapk12, Gata3, Jun, Maf |
| Inflammatory bowel disease (IBD) | 9 | 58 | 0.86 | 0.0013 | Il12rb1, Il4, Il13, Il12a, Il4ra, Rora, Jun, Maf, Foxp3 |
| Th17 cell differentiation | 10 | 100 | 0.67 | 0.0069 | Il12rb1, Il4, Il4ra, Rora, Jak2, Mapk12, Gata3, Jun, Foxp3, Ahr |
| Fc epsilon RI signaling pathway | 8 | 66 | 0.75 | 0.0081 | Il4, Csf2, Il13, Vav3, Lcp2, Pla2g4f, Alox5ap, Mapk12 |
| T cell receptor signaling pathway | 9 | 100 | 0.62 | 0.0208 | Il4, Csf2, Ctla4, Vav3, Lcp2, Mapk12, Jun, Itk, Cblb |
| Pathways in cancer | 24 | 522 | 0.33 | 0.0215 | Cdh1, Pparg, Il12rb1, Il4, Smo, Wnt10a, Runx1t1, Il13, Epas1, Mgst3, Notch1, Il12a, Il4ra, Gsta4, Dapk2, Frat2, Jak2, Ep300, Dapk1, Jun, Plcb1, Bcl2l11, Col4a3, Cdk6 |
