# Supplementary material for: The Differentially Expressed Genes Responsible for the Development of T Helper 9 Cells From T Helper 2 Cells in Various Disease States: Immuno-Interactomics Study
Source: JMIR Bioinform Biotechnol. 2023 Feb 23;4:e42421. doi: 10.2196/42421 (PMC11135241; doi:10.2196/42421)
Supplement: Multimedia Appendix 2 [file bioinform_v4i1e42421_app2.docx]

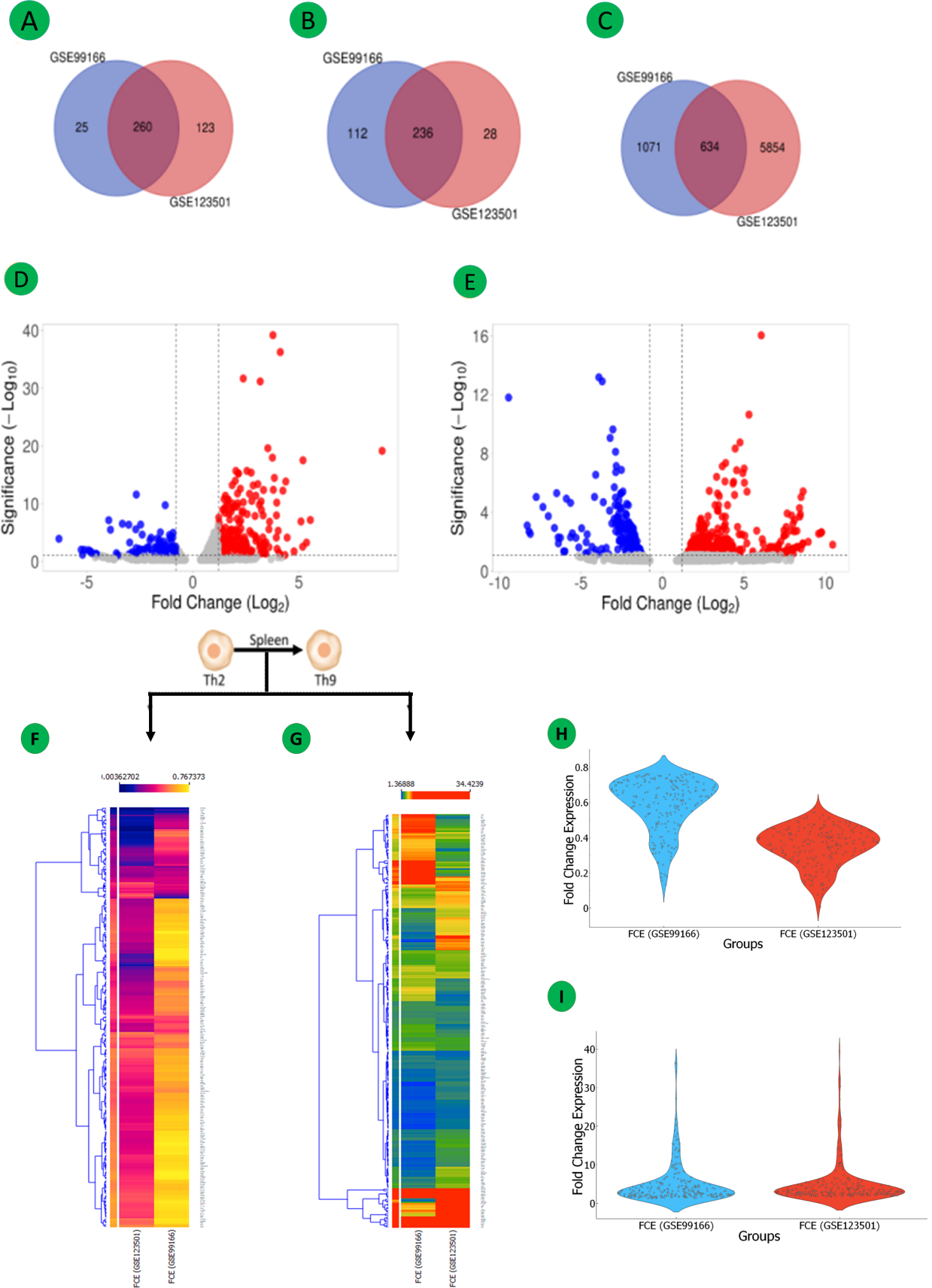


**Figure 3.** Differential mRNA expression of the 2 data sets (GSE99166 and GSE123501) for Th2 to Th9 differentiation: Venn diagrams (A-C) of the 3 data sets' total common, downregulated, and upregulated DEGs; volcano plot for the data sets GSE99166 (D) and GSE123501 (E); and heat maps of common downregulated (F) and common upregulated (G) DEGs extracted from the data sets consisting of genes from Th2 and Th9 cells. FCE levels are displayed in ascending order from blue to yellow. Violin plots (H and I) showing FCE distribution of both data sets for common upregulated and common downregulated DEGs. DEG: differentially expressed gene; FCE: fold change expression; mRNA: messenger RNA; Th: T helper.


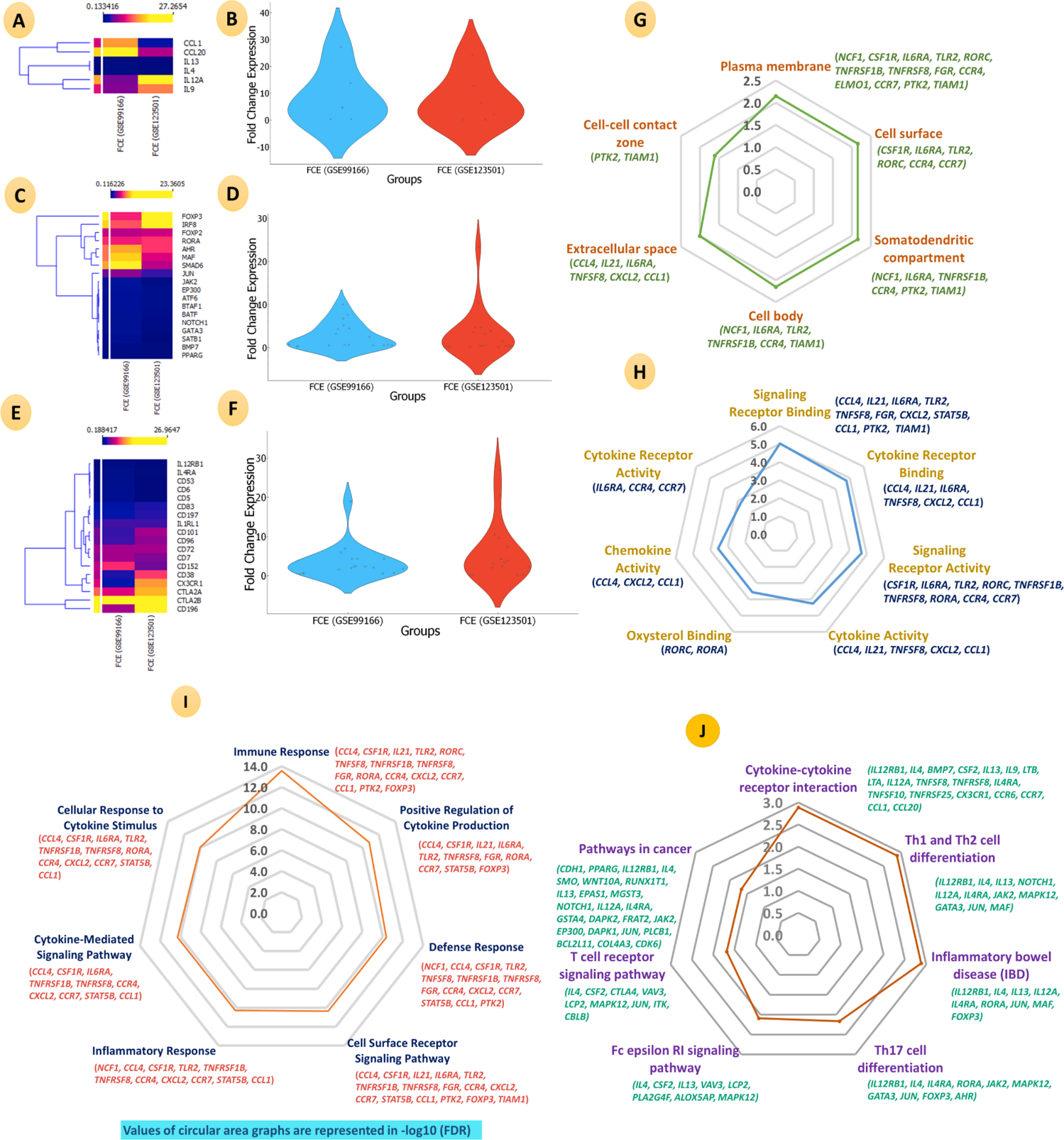


**Figure 4.** Differential mRNA expression of the 2 data sets (GSE99166 and GSE123501) for Th2 to Th9 differentiation: (A, B) interleukin, cytokines/chemokines; (C, D) immune regulating transcription factor; (E, F) immune receptor heat maps and violin plot of all, downregulated, and upregulated DEGs; (G, J) enrichment analysis of common DEGs; (G) Gene Ontology cellular component terms; (H) Gene Ontology molecular function terms; (I) Gene Ontology biological process terms; and (J) the KEGG pathway. The radar plot or circular area graph values are represented in the -log10 (FDR). DEG: differentially expressed gene; FCE: fold change expression; FDR: false discovery rate; KEGG: Kyoto Encyclopedia of Genes and Genomes; mRNA: messenger RNA; Th: T helper.


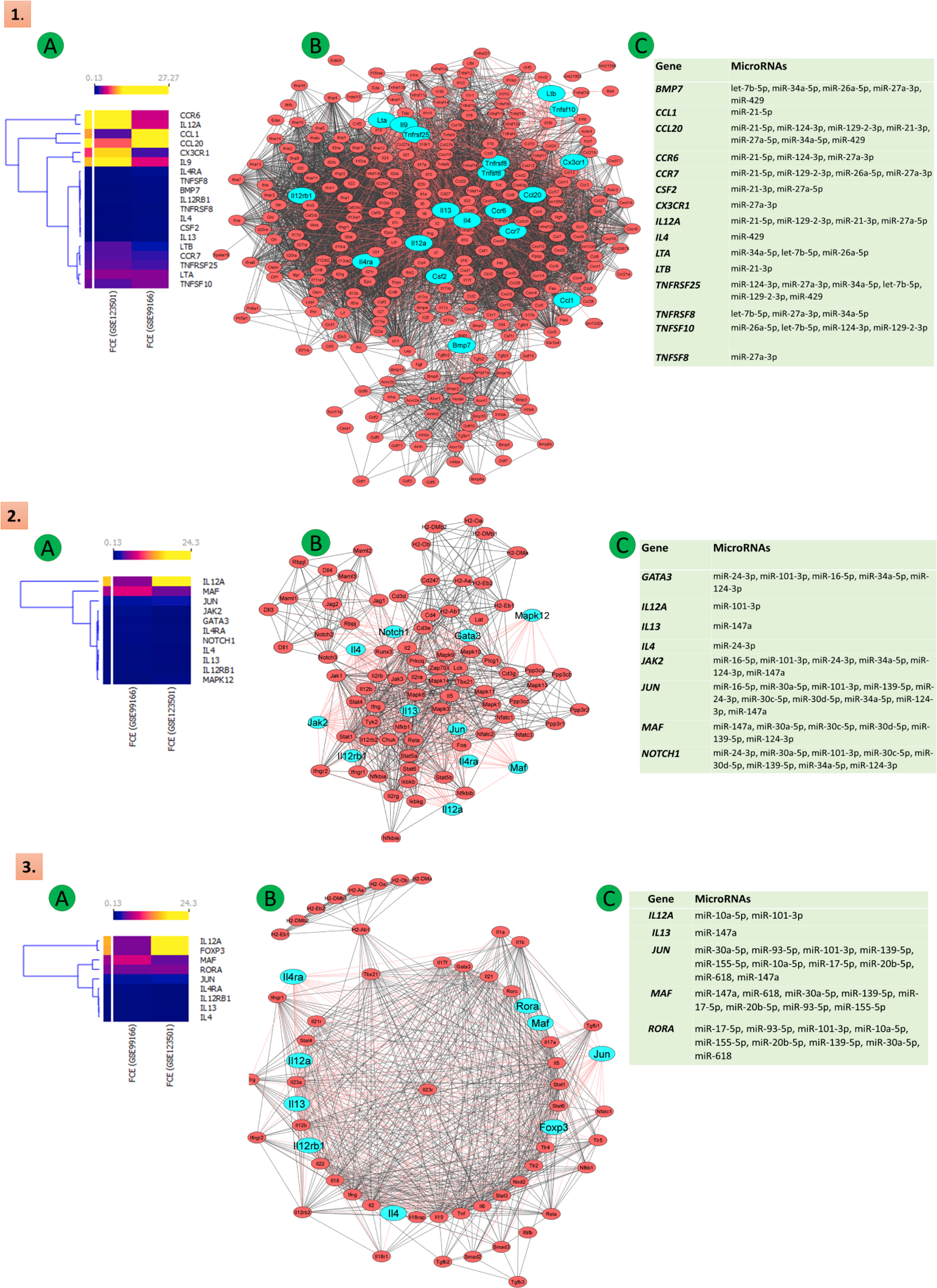


**Figure 5.** Illustration of the Th2 to Th9 differentiation, mainly the 7 pathways involved in this regulatory mechanism: (A) heat maps expression (the upper section of the heat map shows FCE values represented by varying color densities); (B) PPIs networks (top significant DEGs of the network are illustrated in cyan); (C) common posttranscriptional regulatory microRNA pathways—(1) cytokine-cytokine receptor interaction, (2) Th1 and Th2 cell differentiation, and (3) inflammatory bowel disease. FCE: fold change expression; PPI: protein-protein interaction; Th: T helper.


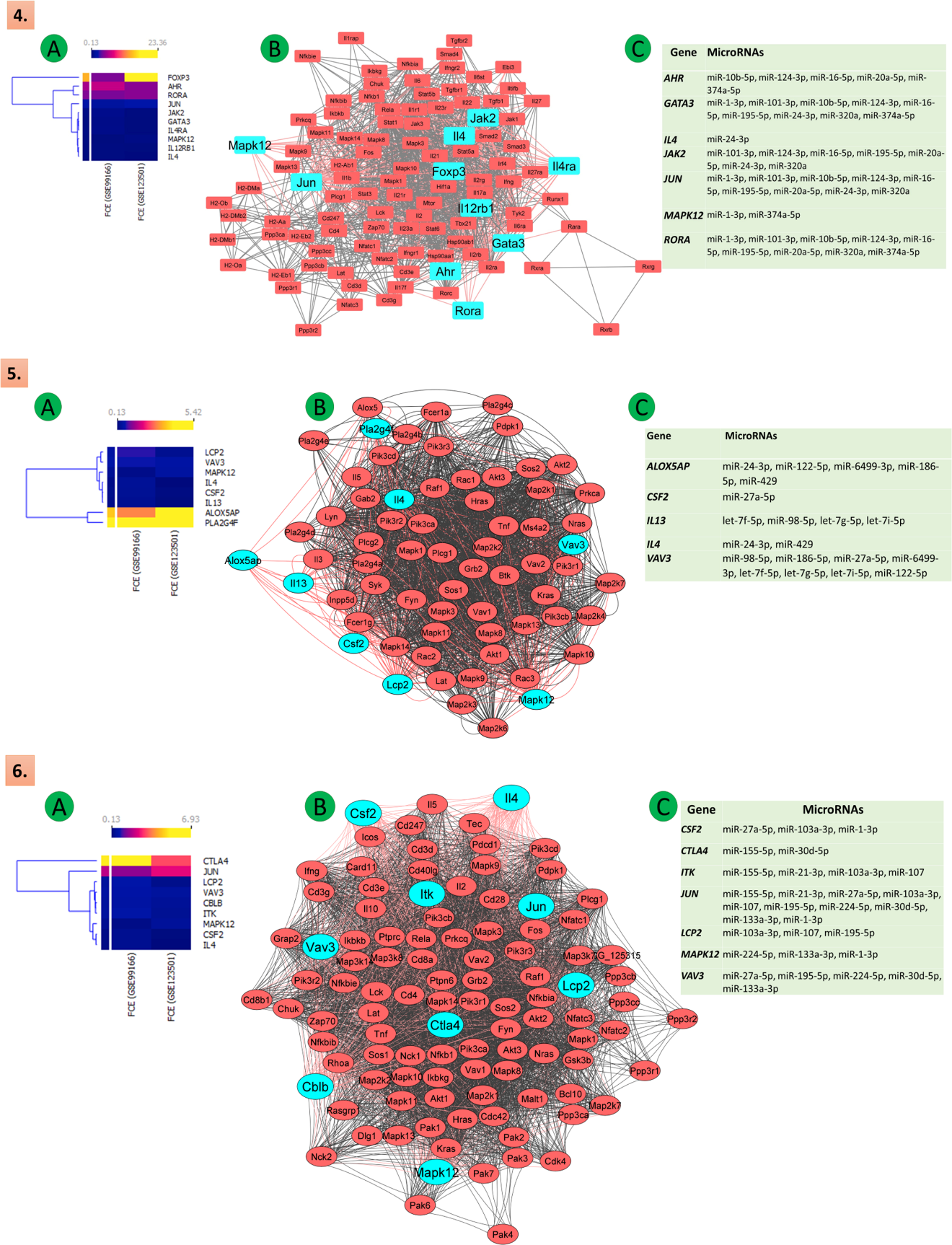


**Figure 6.** Illustration of the Th2 to Th9 differentiation, mainly the 7 pathways involved in this regulatory mechanism: (A) heat maps expression (the upper section of the heat map shows FCE values represented by varying color densities); (B) PPIs networks (top significant DEGs of the network are illustrated in cyan); (C) common posttranscriptional regulatory microRNA pathways (4) Th17 cell differentiation, (5) Fc epsilon and RI pathway, (6) T-cell receptor signaling pathway. DEG: differentially expressed gene; FCE: fold change expression; PPI: protein-protein interaction; Th: T helper.


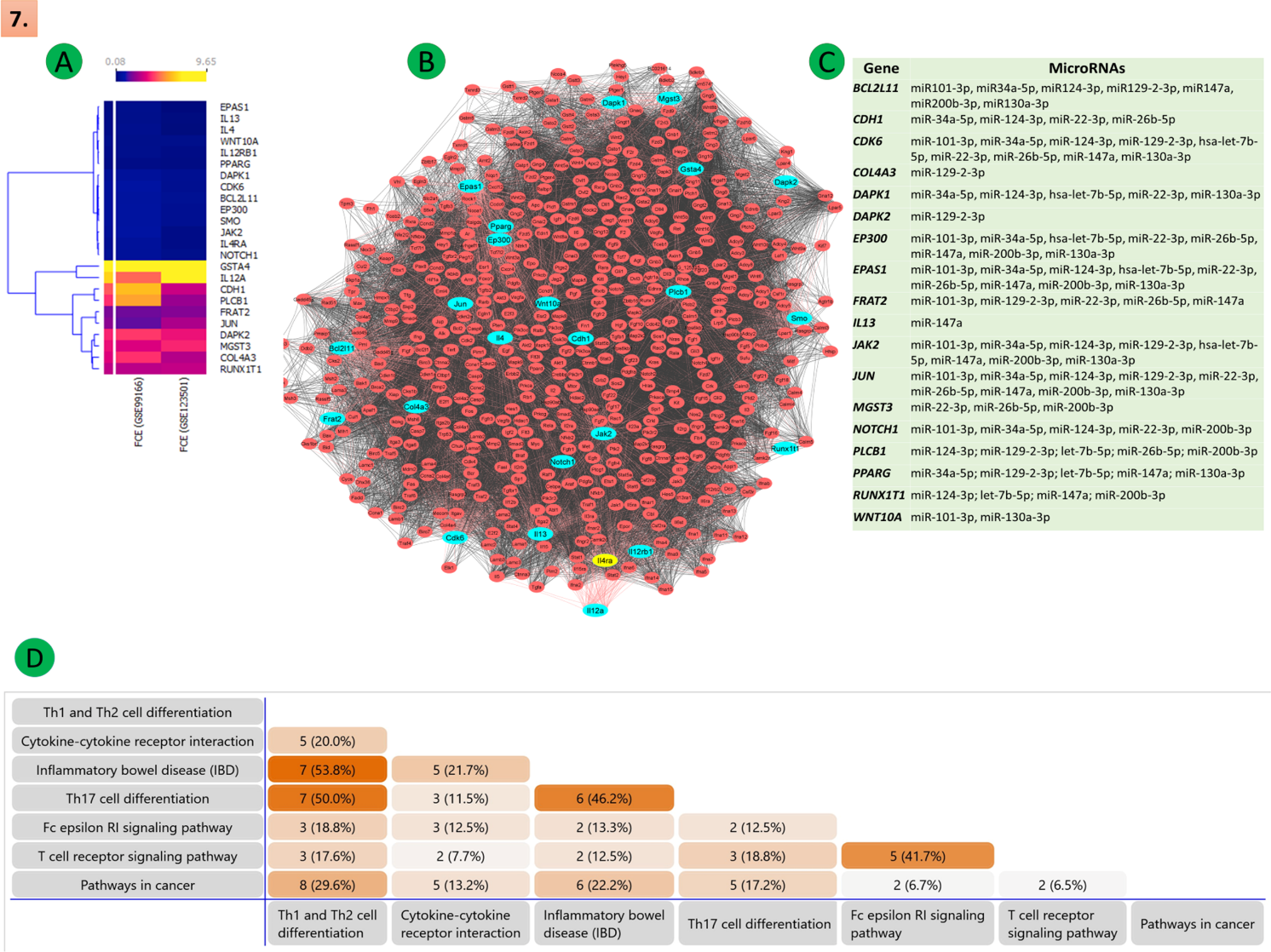


**Figure 7.** Illustration of the Th2 to Th9 differentiation, mainly the 7 pathways involved in this regulatory mechanism: (A) heat maps expression (the upper part of the heat map shows FCE values represented by varying color densities); (B) PPIs networks (top significant DEGs of the network are presented in cyan); (C) common posttranscriptional regulatory microRNA pathways; (D) gene or DEGs similarity in regulatory pathways. DEG: differentially expressed gene; FCE: fold change expression; PPI: protein-protein interaction; Th: T helper.
